# Extracellular vesicles as carriers of mRNA: Opportunities and challenges in diagnosis and treatment

**DOI:** 10.7150/thno.93115

**Published:** 2024-03-11

**Authors:** Robert D. Kirian, Darby Steinman, Christopher M. Jewell, Hannah C. Zierden

**Affiliations:** 1 Fischell Department of Bioengineering, University of Maryland, College Park, MD, 20742; 2 Department of Veterans Affairs, VA Maryland Health Care System, Baltimore, MD, USA; 3 Department of Chemical & Biomolecular Engineering, University of Maryland, College Park, MD, 20742; 4 Robert E. Fischell Institute for Biomedical Devices, College Park, MD 20742, USA; 5 Department of Obstetrics, Gynecology and Reproductive Sciences, University of Maryland School of Medicine, Baltimore, MD, 21201

**Keywords:** extracellular vesicles, biomarkers, mRNA delivery, vaccines, therapeutics

## Abstract

Extracellular vesicles (EVs) are produced by all cells in the body. These biological nanoparticles facilitate cellular communication through the transport of diverse cargoes, including small molecules, proteins, and nucleic acids. mRNA cargoes have gained particular interest given their role in the translation of functional proteins. As a biomarker platform, EVs can be found in nearly all biofluids—blood, mucus, urine, cerebrospinal fluid, and saliva—providing real-time insight into parent cell and tissue function. mRNAs carried by EVs are protected from degradation, resulting in improved detection compared to free mRNA, and recent work demonstrates promising results in using these mRNA cargoes as biomarkers for cancer, neurological diseases, infectious diseases, and gynecologic and obstetric outcomes. Furthermore, given the innate cargo carrying, targeting, and barrier crossing abilities of EVs, these structures have been proposed as therapeutic carriers of mRNA. Recent advances demonstrate methods for loading mRNAs into EVs for a range of disease indications. Here, we review recent studies using EVs and their mRNA cargoes as diagnostics and therapeutics. We discuss challenges associated with EVs in diagnostic and therapeutic applications and highlight opportunities for future development.

## 1. Introduction

**Figure 1:**
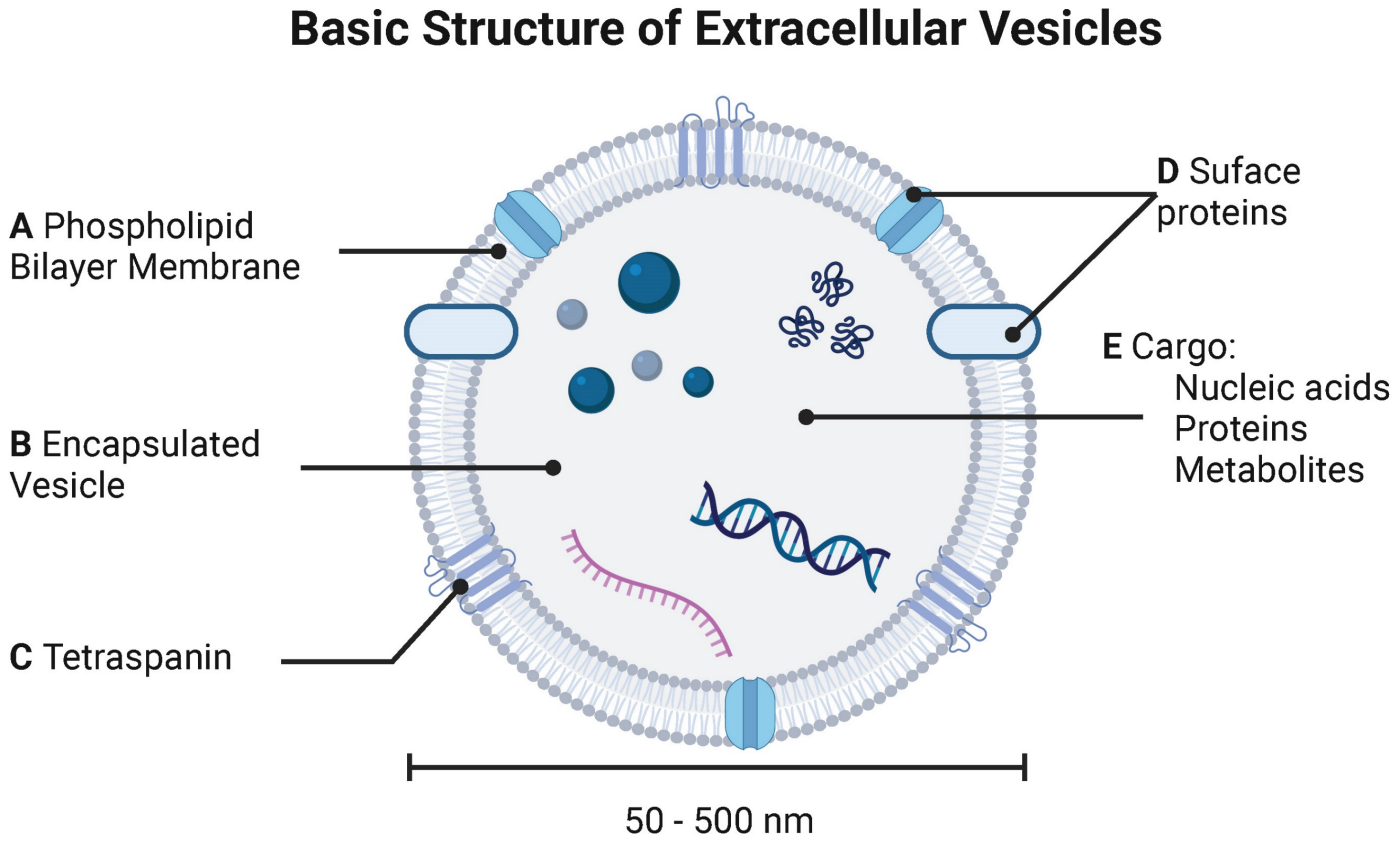
** Basic structure of extracellular vesicle.** EVs are characterized by (**A**) phospholipid bilayer membrane bound (**B**) vesicle decorated by both (**C**) tetraspanins and (**D**) surface proteins. (**E**) EVs carry various cargoes including nucleic acids (DNA and mRNA), proteins, and metabolites.

Extracellular vesicles (EVs) are cell-derived lipid-bound nanoparticles that enable cell-cell communication through transport and delivery of nucleic acids, proteins, and small molecules (**Figure 1**) 1. Due to their natural composition and properties, EVs offer significant potential for identifying and treating a variety of diseases. Throughout this review, we use “EV” as a generic term for all “particles naturally released from cells that are delimited by a lipid bilayer and cannot replicate”, as defined by the International Society for Extracellular Vesicles. The term EV refers to exosomes, microvesicles, and apoptotic bodies released by mammalian cells, as well as outer membrane vesicles, outer-inner membrane vesicles, explosive outer membrane vesicles, and cytoplasmic membrane vesicles released by bacteria 2, 3. In this section, we provide a high-level overview of types of EVs, standard practices for EV isolation, and common approaches for characterization of EVs and their cargoes.

In mammalian cells, exosomes (50-150 nm) are generated as endosomes and produced from the invagination of multivesicular bodies 4. Alterations of the endosomal membranes, addition of surface membrane proteins, and select cargo loading results in the production and release of exosomes 4-6. Microvesicles (100 - 1,000 nm) are produced by the plasma membrane in healthy cells, while apoptotic bodies (100 - 5,000 nm) bleb from the plasma membrane during cell death 5-10. Gram-negative bacteria produce outer membrane vesicles (20-300 nm) and outer-inner membrane vesicles (60-160 nm) through membrane blebbing. Explosive outer membrane vesicles (20-250 nm) are generated through explosive cell lysis 11, 12. Gram-positive bacteria produce cytoplasmic membrane vesicles (20-400 nm) during cell death or lipoprotein distribution 13. During EV biogenesis, RNAs and other biological cargoes are both passively and actively loaded. Molecules found in the cytosol may become passively encapsulated in lipid membranes during EV production. Alternative hypotheses suggest that specific RNA sequence motifs interact with lipid membranes to facilitate loading during EV production, or that RNAs interact with proteins involved in EV loading 14-17. Although the mechanistic biogenesis of EVs is well understood, further work must be done to understand the cellular regulation of EV formation and cargo packaging.

A critical aspect of using EVs as biomarkers or as therapeutic agents is isolating pure populations of EVs, which can be difficult due to the presence of similarly sized high- and low-density lipoproteins, ribonucleoproteins, protein aggregates and cell debris in biological samples. Common techniques for EV isolation include ultracentrifugation, size-exclusion chromatography, asymmetric field-flow fractionation, and immunoaffinity purification. Ultracentrifugation relies on the principles of sedimentation, with cell debris cleared at lower centrifugation speeds, followed by isolation of pure populations of EVs at high centrifugation speeds. However, ultracentrifugation typically results in a much lower yield of isolated particles than other techniques. While preclinical studies use ultracentrifugation to isolate EVs, this may be impractical in clinical and commercial settings due to the large and costly equipment needed, the time necessary to isolate EVs, and the low-throughput 18-20. Despite potential drawbacks, ultracentrifugation remains an industry standard due to the simplicity of protocols, the maintenance of EV stability, and the ability to easily calculate the parameters required to obtain the desired diameter of particles 19, 21.

Other approaches to EV isolation include the use of commercially available precipitation kits. These methods reduce the solubility of EVs, allowing them to be easily separated while minimizing EV degradation, increasing throughput, and reducing cost. However, precipitation methods often result in lower purity isolations, with contaminants such as lipoproteins being precipitated with EVs 22. Size-exclusion chromatography (SEC) maintains EV stability in a cost-effective, but low-throughput manner 23. As products are isolated based on size, proteins and protein aggregates may elute in the same fraction as EVs, limiting the purity of samples. Asymmetric field-flow fractionation applies a perpendicular force to a fluid suspension under non-uniform laminar flow conditions, separating heterogenous particles based on size 24. This technique reduces the shear stress experienced by the EVs, while allowing for precise isolation of particles of desired size 24, 25. However, fractionation is unable to handle large sample volumes, limiting the scalability 25. Immunoaffinity purification uses targeting ligands, conjugated to magnetic beads that bind to surface proteins expressed on EVs to separate nanoparticles based on affinity 26. Although immunoaffinity purification results in high yield of EVs, it comes with a high cost, limiting large scale usage 25. ELISA-based immunocapture approaches may increase the yield of EVs, however background noise and non-specific binding require optimization for specific membrane proteins, limiting widespread usage 20, 27. Direct comparisons of EV isolation techniques reveal clinically relevant differences that should be considered when using EVs as biomarkers and therapeutics. For example, SEC results in smaller EVs with higher concentrations of lipoproteins, compared to ultracentrifugation, whereas dialysis may decrease protein and RNA contents compared to SEC or ultracentrifugation 28-30. Ultimately, EV isolation technologies must work towards high-throughput, highly reproducible, and cost-effective methods to fully enable clinical translation of EVs as biomarkers and as therapeutics.

Once EVs are isolated, typical characterization includes assessing the number of EVs and the particle size distribution in a sample. The most common methods used to measure concentration and size rely on the Brownian motion of particles in solution. In the same medium, small particles move more vigorously than large particles. Using the Stokes-Einstein relationship for diffusion, the approximate hydrodynamic radius of particles can be calculated based on the scattered light intensity of a bulk sample, as in dynamic light scattering (DLS), or based on each particle's individual motion, as in nanoparticle tracking analysis (NTA). Direct comparisons of NTA vs. DLS measurements on cerium oxide nanoparticles showed that DLS readings were skewed due to the presence of large particles or aggregates 31. Isolated EVs are prone to aggregation, which may cause similar issues with DLS. In contrast, NTA can distinguish particle sizes and determine an overall size distribution, improving the usefulness of resulting data 31.

Less common approaches include characterization based on EV membrane proteins 32. The use of specific antibodies allows for identification of EVs from specific parent cells, but often fails to give a complete picture of EV composition in a sample. To capture an entire population of EVs, multiple surface markers can be used, but this increases overall expense and is relatively low-throughput 33. Lipophilic and other complete membrane dyes may be used, however, these may alter EV properties, decrease stability, or increase particle aggregation 34, 35. Flow cytometry technologies have been expanded to the EV field, however, unbound dye may limit signaling from EVs resulting in low output readings after normalization 36. As characterization technologies continue to expand, researchers should closely follow the guidelines published by the International Society for Extracellular Vesicles regrading EV quantification and characterization. Adherence to these guidelines will help to standardize EV work and accelerate the clinical translation of EVs as biomarkers and therapeutic carriers 3.

Just as high throughput isolation and characterization technologies for EVs have been developed, so have technologies used to analyze the cargo of EVs such as lipids, proteins, and nucleic acids. As these cargoes reflect parent cell function, accurate and thorough characterization allows for use of EVs as biomarkers of cellular dysregulation and disease. Recent focus has turned towards characterizing messenger RNA (mRNA) cargoes given their use as early biomarkers of disease. mRNA carries the coding sequence essential for the translation of functional proteins, and differences in circulating mRNA may highlight protein dysregulation in a diseased state. Further, mRNAs carried by EVs may provide an improved biomarker compared to free mRNA due to the protection provided by the lipid membrane 37. Traditional assays for analyzing EV nucleic acid cargoes include microarrays, Northern blot, sequencing, and reverse transcription polymerase chain reaction (RT-PCR) 38. Emerging work has used clustered regularly interspaced short palindromic repeats (CRISPR)-based assays, nanoparticle-based biochips, electrochemical biosensors, surface-enhanced Raman spectroscopy, and surface plasmon resonance 38. As the use of EVs continues to grow, the development of effective, reproducible, and high-throughput isolation and characterization technologies will advance clinical applications of EVs.

This review explores both the diagnostic and therapeutic potential of mRNAs carried by EVs. We review techniques used to isolate EVs from biofluids, examine strategies to load mRNA into EVs, and identify current applications of EVs in diagnosing and treating a spectrum of diseases. We also discuss challenges facing the EV field and highlight opportunities for future work.

## 2. mRNA cargoes as biomarkers of disease

The clinical diagnostics market is valued at nearly 80 billion USD and is expected to increase to over 100 billion USD by 2028 39. Current diagnostic approaches involve imaging (MRI, CT, ultrasound), biopsies, and/or bloodwork, in conjunction with patient symptoms. Advances in imaging techniques involve the use of radioactive particles to improve resolution and to identify specific cell-types 40. Despite improvements in imaging technologies, these approaches are often costly, and require additional tissue or blood biopsies to achieve high confidence diagnoses. In many cases, the timing of diagnosis can dramatically alter disease progression, with early intervention increasing survival rates. However, many indications present with variable symptoms, heterogeneous pathologies, and unclear etiologies, making early diagnosis difficult 41.

Recent advances in point-of-care devices have improved clinical abilities to assess circulating metabolites, hormones in urine, and the presence of blood in fecal matter. These devices enable rapid results and reduce cost and labor requirements by eliminating the need to send samples to specialized laboratories for processing. However, reagents often require low temperature conditions to maintain stability, limiting widespread usage 42. Diagnostic ELISAs can detect antibodies or antigens in biofluids, improving sensitivity to specific biomarkers, but require large processing times and are typically unable to detect nucleic acid biomarkers. DNAs and RNAs are subject to degradation throughout the body but give high resolution information about variations in the genome and transcription status of various tissues. To improve the accessibility of nucleic acids as circulating biomarkers, recent interest has turned to the diagnostic potential of EVs as they offer a means to improved assessment of circulating mRNA cargoes. In contrast to free mRNA, mRNA EV cargoes are protected from degradation, allowing for the analysis of mRNAs in higher concentrations and improving the usage of mRNAs as biomarkers which reflect parent cell function. In this section, we discuss recent work done to establish EV-based biomarkers (**Table 1**), mRNA-based biomarkers (**Table 2**), and mRNAs carried by EVs as biomarkers of a spectrum of diseases (**Table 3**).

### 2.1 EVs and mRNA cargoes as biomarkers for cancer

Cancer diagnosis can be a long and invasive process. Evaluating mRNA cargoes carried by plasma-derived EVs may be more cost- and time- effective than current biopsy and imaging methods. For example, growing rates of colorectal cancer emphasize the need for detection methods which enable earlier treatment. In an effort to improve blood-based biomarkers of colorectal cancer, plasma-derived EVs have been investigated, revealing similarities between plasma-derived EVs from patients with colorectal cancer and media-derived EVs from cultures of colorectal cancer cells 43. Both blood- and culture-derived EVs showed high concentrations of mRNAs involved in the pathogenesis of colorectal cancer through regulation of cell growth, proliferation, migration, and angiogenesis pathways 43. Independent of EVs, free Metadherin mRNA, a key regulator in the MAPK pathway responsible for tumor progression and metastasis, was identified as a biomarker of colorectal cancer, allowing for differentiation of tumor stages 44. Future work may look at EV-carried Metadherin as a more refined biomarker of disease.

Plasma-derived EVs have also been investigated as biomarkers for prostate cancer. One major challenge with the treatment of prostate cancer is cell resistance to testosterone-lowering treatments, which results in worsened prognoses and rapid tumor progression. Plasma-derived EVs and mRNA cargoes may indicate resistance to treatment, with increased levels of AR-V7, a prostate-cancer specific mRNA, being found in circulating EVs from patients who responded poorly to testosterone-lowering treatments 45.

While plasma is an obvious biofluid for EV isolation and analysis, local biofluids may provide access to higher concentrations of EVs from relevant tissues. The progression of non-small cell lung cancer, for example, may be reflected in respiratory mucus. EVs from bronchoalveolar lavage from both smokers and patients with non-small cell lung cancer carried increased levels of mRNAs involved in proliferation, differentiation, and maintenance of cancer stem cells, as compared to healthy controls 46. Similarly, urine-derived EVs may be used as biomarkers of bladder cancer if they are found to carry increased levels of proteins involved in cell adhesion, motility, cell survival, and proliferation 47. A further look into mRNA cargoes in EVs isolated from local biofluids may provide more relevant and improved biomarker detection.

### 2.2 EVs and mRNA cargoes as biomarkers for infectious diseases

The recent coronavirus disease 2019 (COVID-19) pandemic highlighted several challenges associated with the diagnosis of infectious diseases. Positive diagnoses are often limited by incubation times, when the host has been infected, but the pathogen has not yet replicated to measurable levels. Furthermore, the range of individual patient responses to infectious diseases makes it difficult to identify appropriate treatment regimens. Recent work identified blood-based biomarkers as predictors of COVID-19 prognosis, where an increase in COPB2, a protein responsible for vesicle transport, was associated with milder symptoms and swifter recovery 48, 49. Although this study did not specifically investigate EVs, it motivates future work to understand EVs and cargoes as biomarkers of disease severity, where the sensitivity of diagnostic assays may be improved using EVs and mRNA cargoes.

The COVID-19 pandemic especially impacted immunocompromised patients and patients with underlying health conditions. For example, people living with human immunodeficiency type-1 virus (HIV-1) have higher rates of underlying conditions due to impaired immune function and chronic inflammation. While antiretroviral treatments have improved the prognosis of people living with HIV-1, patients are at an increased risk for morbidities, including metabolic, cardiovascular, and neurodegenerative disorders 50. Biomarkers to predict these morbidities may help to improve patient quality of life. EVs produced by HIV^+^ cells may contribute to chronic inflammation by triggering a pro-inflammatory cytokine cascade 50, 51. One study found an increased concentration of plasma-derived EVs from HIV-1 patients with detectable viral loads 52. EVs were found to be larger in size with an increased concentrations of DAP3, a protein indicative of apoptosis 52. Future work could investigate mRNA cargoes as indicators of chronic inflammation with implications for co-morbidities associated with HIV infections.

Tuberculosis is a deadly infection caused by *Mycobacterium tuberculosis.* Latent tuberculosis affects a quarter of the population, and despite individuals exhibiting no symptoms, they are at risk for future tuberculosis infections 53. While treatment of latent tuberculosis decreases infectivity, current diagnostics are dependent on host immune response to *M. tuberculosis* antigens 54, 55. Recent work has turned towards developing minimally invasive blood-based assays to identify patients with latent tuberculosis. Compared to healthy participants, serum-derived EVs from individuals with latent tuberculosis carried tuberculosis-associated peptide cargoes involved in glutamine metabolism, nitrogen metabolism, and protein stability, suggesting EVs may be useful as biomarkers of latent tuberculosis as well as other infectious diseases 56.

### 2.3 EVs and mRNA cargoes as biomarkers for neurological disease

Neurodegenerative diseases such as Alzheimer's disease and Parkinson's disease develop long before clinical symptoms occur. Once symptoms manifest, these diseases are often too progressed for effective and long-term treatments 57. However, mRNA cargoes from circulating EVs may allow for earlier detection and earlier intervention 58, 59.

**Figure 2:**
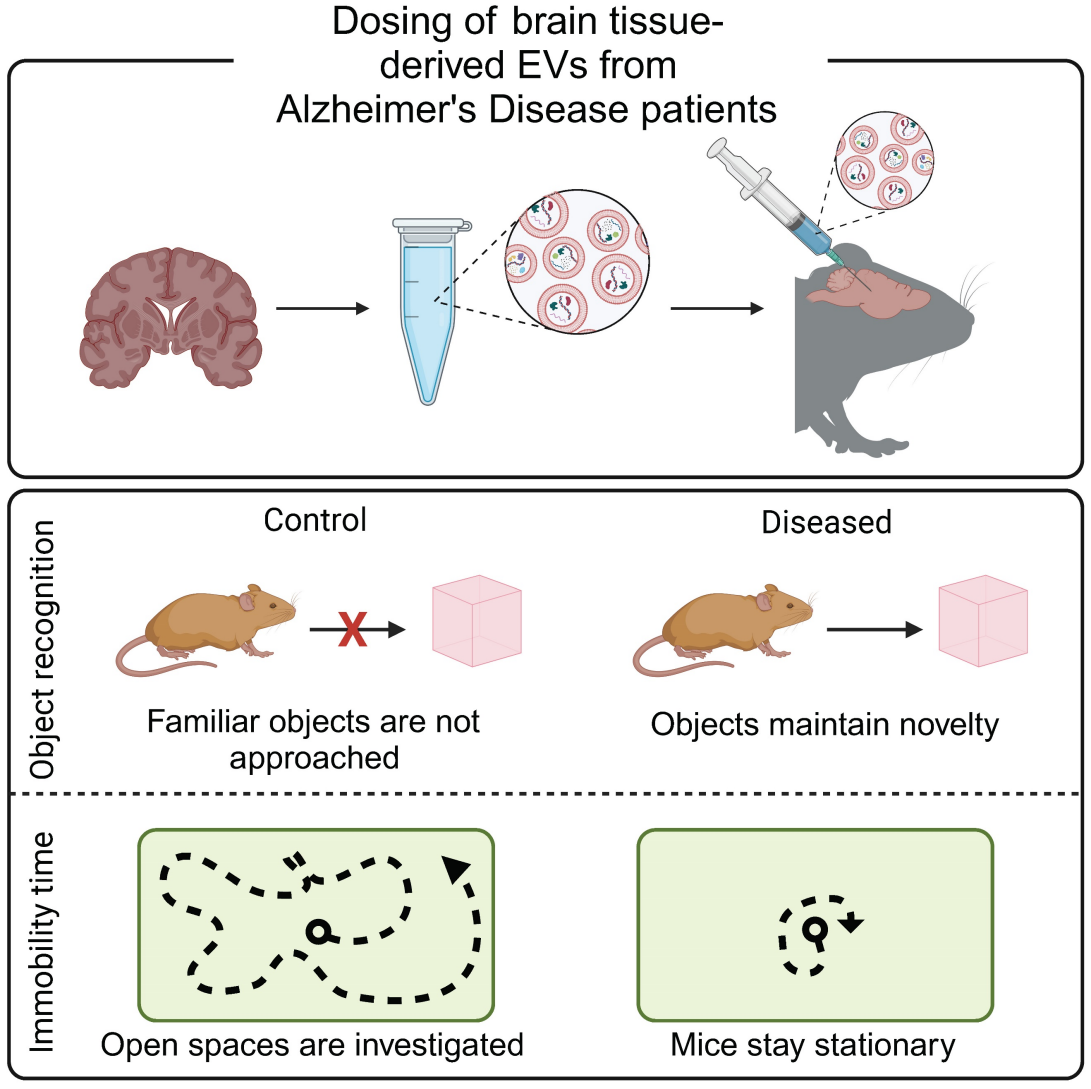
** Brain-derived EVs result in Alzheimer's-like cognitive impairment *in vivo.*** EVs were isolated from *post mortem* brain tissue from Alzheimer's disease patients and injected into the hippocampus of mice. Mice demonstrated decreased mobility during open field tests and increased exploration time during novel object recognition tests, reflective of Alzheimer's disease phenotypes. Adapted with permission from 61, copyright 2023 John Wiley & Sons, Inc.

Alzheimer's disease begins with the accumulation of β-amyloid in the brain, which negatively impacts the longevity of neurons. An accurate diagnosis must wait until the accumulation of β-amyloid plaques, or the corresponding neuron deterioration, reaches an imaging threshold or results in a cognitive deficient 57. An alternative diagnostic approach analyzes cerebrospinal fluid for evidence of β-amyloid and aggregated tau. However, lumbar punctures often cause nausea, vomiting, irritation of nerves, or bleeding into the spinal canal 60. EVs have been hypothesized to contribute to Alzheimer's disease progression, making them ideal biomarkers of early disease. In preclinical studies, mice dosed with EVs derived from *post mortem* brain-tissue from patients with Alzheimer's disease exhibited Alzheimer's disease-like behaviors (**Figure 2**) 61. Similarly, EVs derived from an *in vitro* model of Alzheimer's disease increased tau phosphorylation when administered to mice 62. Together, these studies support the contribution of EVs to Alzheimer's disease progression and suggest that EVs may serve as an early biomarker of disease. Indeed, plasma-derived EVs from patients with Alzheimer's disease and patients with mild cognitive impairments contained increased concentrations of mRNA as compared to healthy controls 63. Similarly, brain-tissue derived EVs from a mouse model of Alzheimer's disease contained increased levels of β-amyloid precursor protein, upregulation of a presenilin protein, and downregulation proteins associated with protein folding and RNA synthesis as compared to healthy controls 64.

Parkinson's disease is another neurological condition with few symptoms in early stages of disease. Although recent work found no significant differences between plasma-derived EVs from healthy and Parkinson's disease groups, an increase in EV-associated tau was associated with a decline in cognition within the Parkinson's disease group 65. Investigation into the role of miRNA as a biomarker of Parkinson's disease severity, concluded that variation in the concentration of miRNAs associated with proliferation and differentiation was associated with disease severity 66. Separate from EVs, free long noncoding RNAs (lncRNA), free mRNAs, and free protein are suggested as biomarkers of Parkinson's disease that may contribute differences in the adaptive immune response 67, 68. Combined, these studies suggest that analysis of plasma-derived EVs and corresponding mRNA cargoes may enhance the prediction of disease onset and severity.

Autism spectrum disorder (ASD) is characterized by impaired social and learning development. With diverse etiologies and pathologies, the diagnosis of ASD is often a long process. Some reports suggest it takes an average of 3.5 years for an official diagnosis 69, 70. Circulating EVs may serve as indicators of ASD before behavioral assessments are feasible. Plasma-derived EVs from children with ASD showed differences in several lncRNA and mRNA cargoes as compared to control groups 71. EVs from patients with ASD carried increased concentrations of synaptic vesicle-associated lncRNA transcripts but had non-detectable levels of free synaptic vesicle mRNA associated with ATP dependent transform 71. Plasma-derived EVs from patients with ASD also carried increased levels of total protein as compared to controls 72. In particular, EVs carried increased concentrations of neurotensin, which has been shown to increase IL-1β, a cytokine associated with ASD 72, 73. Peripheral blood mononuclear cells from patients with ASD were found to have lower levels of mRNAs coding for estrogen receptors, estrogen-related receptors, and retinoic acid-related receptors 74. These findings support previous associations between high levels of estrogen in the prenatal window and ASD pathogenesis 75. Peripheral blood samples from male patients with ASD showed increased levels of free mRNA associated with behavioral hallmarks of ASD 76. The same trend was not observed for female patients, highlighting the need for improved biomarkers of ASD in females 76. Another strategy for enhanced detection of ASD focused on L1CAM-positive EVs 77. L1CAM is a neural cell adhesion molecule associated with neurodivergence. In a cohort of male patients, L1CAM-positive plasma-derived EVs carried decreased levels of mRNAs and miRNAs involved in neuroactive ligand-receptor interaction, neurodegeneration, dendrite formation, neuron migration, and the neuron apoptotic process, providing insight into the role of EVs in ASD and highlighting a potential biomarker for earlier ASD detection 77.

In addition to genetic and molecular pathologies underlying neurological conditions, risk for depression and other mental illnesses is impacted by social and psychological factors 78. Current diagnostic assessments use symptomatic questionnaires, but have few molecular biomarkers. Given evidence that saliva-derived EVs may be reflective of brain function, recent work investigated their use as biomarkers for general depression and anxiety disorders 79. The level of M6a protein in EVs was positively correlated with perceived stress scores in patients with depression 79. The authors reported no significant difference between individuals undergoing treatment and the control groups, suggesting that M6a concentration could be used to evaluate the efficacy of antidepression therapies 79. Postpartum depression is associated with increased anxiety, restlessness, loss of appetite, insomnia, and social withdrawal which manifests in mothers towards the end of pregnancy or following childbirth. While some cases are an extension of a previous condition, many patients have no prior history of mental illness 80. Plasma-derived EVs from patients who did not experience postpartum depression demonstrated relatively consistent concentrations of mRNA cargoes throughout pregnancy, whereas EVs from patients who developed postpartum depression exhibited increasing levels of mRNA cargoes during the third trimester, followed by a sharp decline after giving birth. mRNA cargoes involved in autophagy functions were decreased and mRNAs associated with ribosomal and mitochondrial function were increased in the postpartum depression group as compared to the control group, highlighting the potential for mRNA cargoes to serve as indicators of mental health 80.

### 2.4 EVs and mRNA cargoes as biomarkers for gynecologic and obstetric health

Beyond postpartum depression, EVs and their cargoes have shown potential as biomarkers for women's health indications. Combined with a lack of clear pathologies, screening for gynecologic and obstetric disease is difficult 81. As the concentration of circulating EVs dramatically increases during pregnancy, EVs have been suggested as biomarkers of gestational diseases.

Placental dysfunction, due to a lack of cellular differentiation and abnormal implantation into the uterine wall, can manifest as preeclampsia. Preeclampsia is characterized by high blood pressure, headache, changes in vision, nausea, or swelling. Current treatment of preeclampsia is delivery of the fetus, regardless of gestational age. To develop effective interventions, earlier diagnosis must be made possible. Compared to EVs from controls, EVs from patients with preeclampsia had higher concentrations of several proteins, including CRH, which is involved in stress response, and KRT16, which regulates immunity against epidermal barrier breach 82. A separate study found that there were higher concentrations of small EVs (50-150nm) from patients with preeclampsia, and that EVs carried higher levels of IL-21 and TGF-β, compared to the control group 83. The placenta is also implicated in gestational diabetes mellitus (GDM), a condition in which pregnancy and pregnancy-related hormones limit efficient insulin production. GDM is associated with maternal cardiovascular risks, both during and after pregnancy, as well as risk of placental dysfunction. Early diagnosis and treatment of GDM may limit the long-term effects on fetal development. Maternal plasma-derived EVs from patients with GDM showed increased concentrations of miRNA associated with metabolism and inflammation. Future work could investigate mRNA cargoes from circulating EVs in early pregnancy to indicate risk for placental dysfunction later in pregnancy 83.

Beyond predicting placental diseases, it is difficult to determine pregnancies at risk for spontaneous and unexplained miscarriages, which occur in ~26% of pregnancies. Plasma-derived EVs from individuals who suffered a miscarriage contained lower levels of CD9, an EV surface protein, compared to patients who experienced healthy pregnancies 84. Although nucleic acids were not assessed in this study, a shift in membrane proteins highlights potential differences in biogenesis and loading that may result in differences in mRNA cargoes. Preterm birth, defined as delivery before 37 weeks of gestation, occurs in ~10% of all pregnancies 85. One study evaluated EVs and miRNA cargoes over the course of pregnancy, identifying differences in miRNA cargoes associated with normal cell cycle 86. Together, these reports may provide a baseline for normal pregnancies, contributing future studies of biomarkers for atypical pregnancies.

Maternal plasma-derived EVs contain cargoes which may also be useful in diagnosing fetal genetic disorders. Common methods of prenatal testing are invasive and may pose additional risk to the developing fetus. In one study, maternal plasma-derived EVs carried significantly lower concentrations of DNA in cases where fetuses were later diagnosed with chromosomal diseases, demonstrating that EVs may serve as an alternative screening tool 87. A separate study investigated biomarkers for fetal antenatal hydronephrosis, a malformation of the urinary tract 88. Amniotic fluid-derived EVs had significant differences across mRNA and protein cargoes related to antenatal hydronephrosis. Moesin, an mRNA cargo associated with proliferation and epithelial integrity, was upregulated in amniotic fluid EVs from fetuses with fetal antenatal hydronephrosis. Moesin showed better diagnostic potential than current clinical biomarkers, such as ultrasound visualization 88. However, sampling of amniotic fluid requires an invasive procedure with risks of infection, miscarriage, or preterm birth 89, 90. Future work could explore maternal circulating EVs and corresponding mRNA cargoes as biomarkers of fetal chromosomal diseases.

### 2.5 Challenges associated with using EVs as biomarkers of disease

Thus far, we have discussed promising results using EVs as early biomarkers of disease. In this section, we highlight challenges and considerations for the translation of EVs in diagnosing disease and evaluating disease progression.

#### 2.5.1 EV cargoes differ across biological fluids

**Figure 3:**
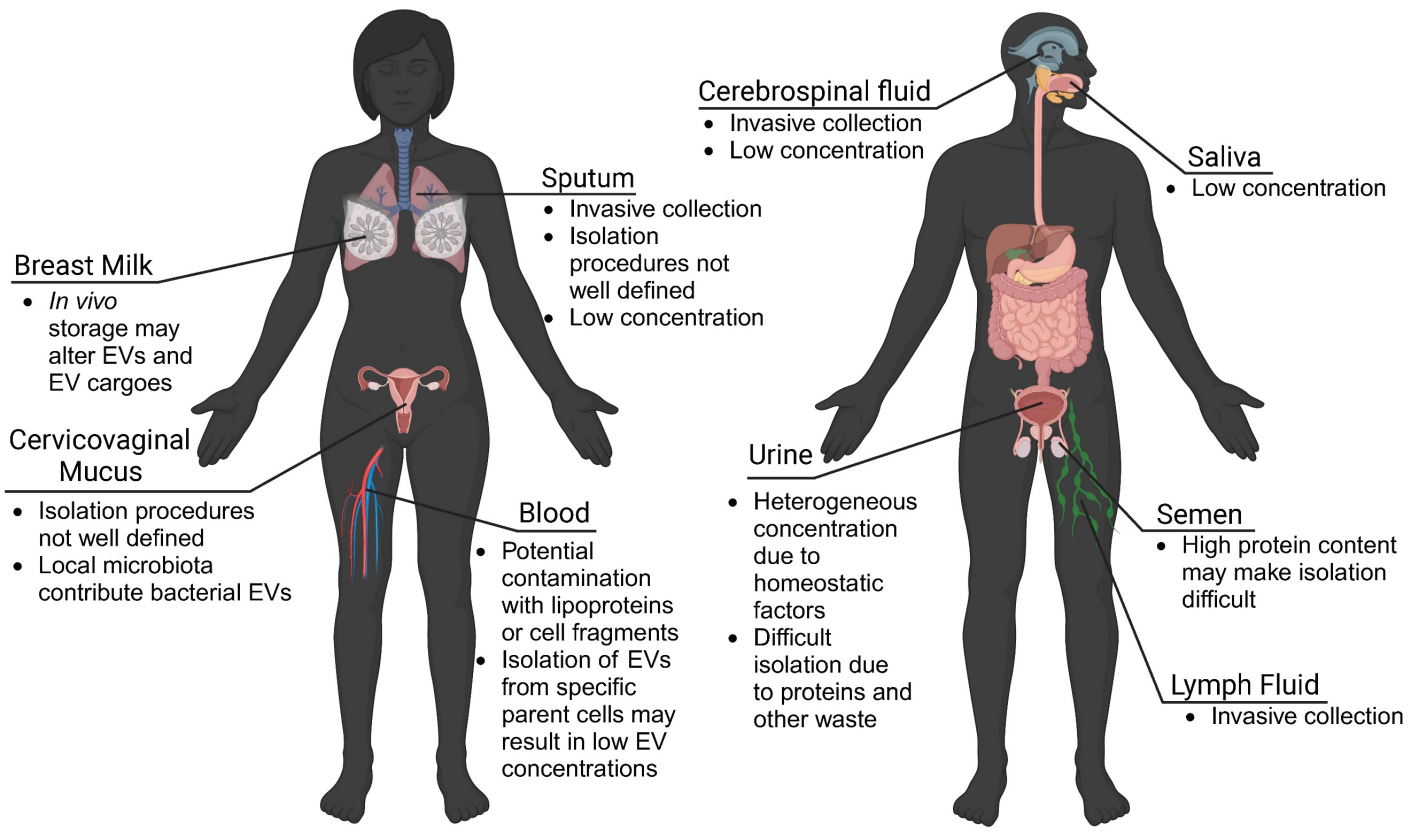
** EVs are found in all biofluids.** While blood plasma is commonly used for EV isolation and biomarker detection, urine-, mucus-, cerebrospinal fluid-, lymph fluid-, and saliva-derived EVs also show potential. However, challenges to collection procedures, isolation, and detection of EVs may limit clinical translation. Advances in isolation and characterization strategies may improve the use of EVs derived from local biofluids as biomarkers of disease.

Using patient-derived EVs and mRNA cargoes as biomarkers may give real-time information about tissue function, decrease the need for invasive tissue biopsies, and improve clinical capabilities of diagnosing disease. Many studies isolate EVs from plasma or serum samples, as routine blood draws and plasma banks provide a large collection of samples to evaluate. However, EVs are found in nearly every biological fluid in the human body, including blood, urine, lymph fluid, saliva, mucus, cerebral spinal fluid, breast milk, and seminal fluid (**Figure 3**). While EVs travel throughout the body, EVs may preferentially accumulate in local biofluids improving biomarker potential. For EVs from these matrices to be translationally relevant, however, there must be technological advances to isolate quantifiable concentrations of EVs.

Urine-derived EVs have been investigated for their role in bladder-associated diseases, and may have increased stability over extended periods 91, 92. Saliva has also been shown to contain measurable concentrations of neural EVs, proving useful for studies on Parkinson's disease, depression, and certain cancers 79, 93, 94. Milk-derived EVs may provide insight into neonatal immune, metabolic, and neurologic development and may be useful for predicting offspring risk for disease 95. EVs found in sputum were shown to contain nucleic acids suitable for genetic analysis, making them potentially useful as biomarkers for pulmonary diseases 96. This suggests that other mucosal surfaces (vaginal, gastrointestinal) may be a source of biomarker EVs. Important to consider though, are unique microbiomes that colonize the vaginal and gastrointestinal environments, which may make isolation of host-derived EVs difficult. Seminal fluid has high concentrations of EVs, and has been utilized for investigations into both male fertility and prostate cancer 97, 98. It is important to consider differences in EVs isolated from different biofluids. For example, in bovine and porcine models, EVs from plasma, cerebral spinal fluid, and seminal fluid, exhibited differences in size and protein content, which may reflect preferential localization of EVs from various parental cells 99. In another comparative study, the isolation of EVs from bovine urine, saliva, and milk suggested limitations in using urine- and saliva-derived EVs as biomarkers due to low EV recovery 95. Similarly, cerebral spinal fluid and lymph fluid contained low concentrations of EVs and associated proteins 99. However, these limitations may be overcome with advances in isolation and characterization technologies.

#### 2.5.2 EVs are affected by homeostasis

**Figure 4:**
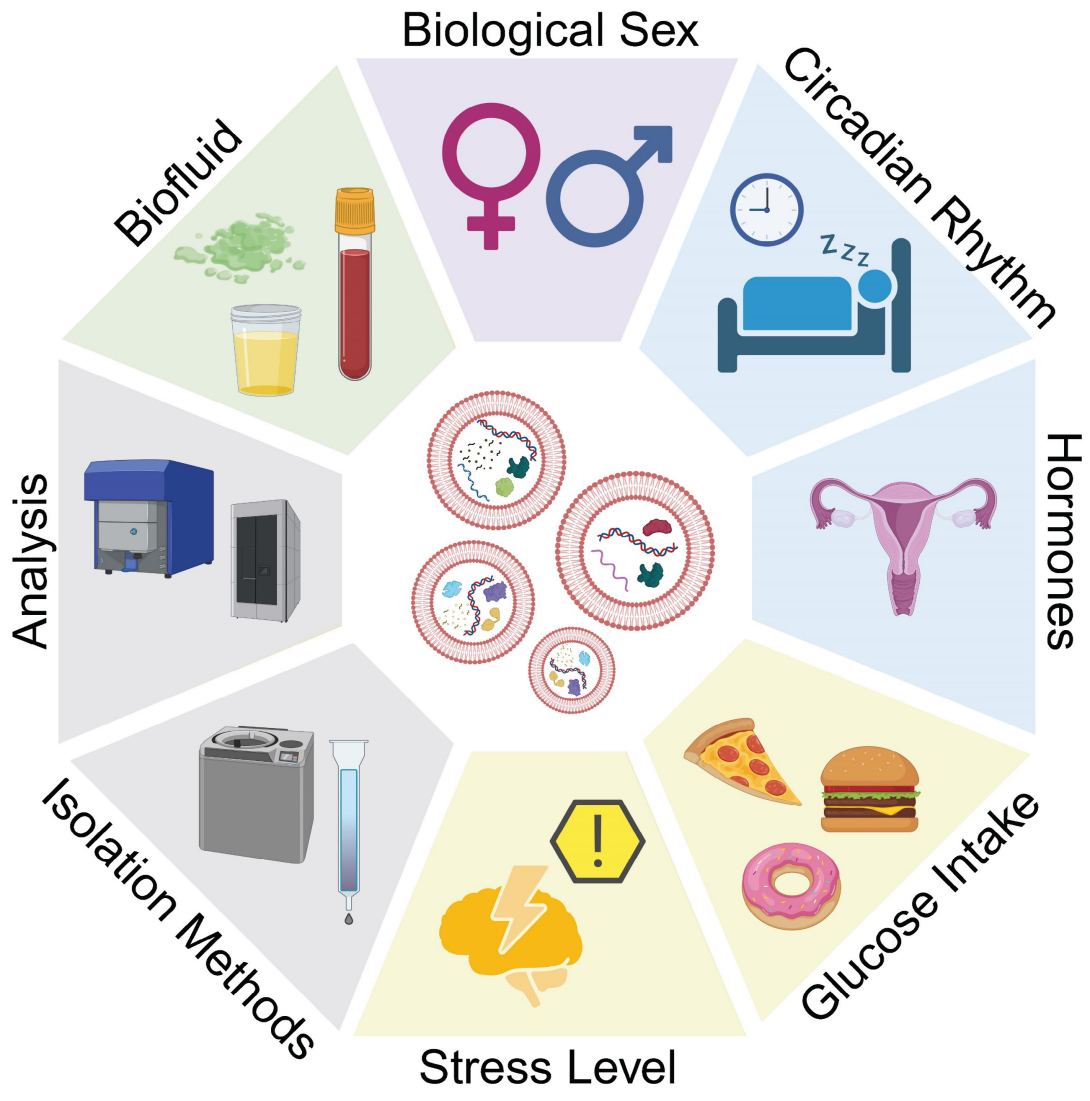
** Considerations for EVs as biomarkers.** Differences in EV size, concentration, and cargo load may be attributed to disease, or other homeostatic variations. Biological factors such as biological sex, circadian rhythm, hormones, food consumption, and stress, as well as isolation methods and characterization protocols may affect experimental observations and must be considered when translating EVs and their mRNA cargoes as biomarkers.

EVs serve as biological communicators in the body, making them an ideal reporter for tissue function, disease severity, and response to treatment. Within an individual, EVs are sensitive to changes in circadian rhythm, hormones, stress, and glucose levels (**Figure 4**). Across patient cohorts, lifestyles and lived experiences may cause variation in EV baselines, which is a further consideration for using EVs as biomarkers of disease. By establishing changes to EV characteristics due to seemingly innocuous perturbations in homeostasis, we may be able to better identify changes to EV characteristics that are reflective of disease state.

The circadian rhythm is a 24-hour behavior cycle which influences sleep, body temperature and appetite in mammals 100. Recent work highlighted the role of the circadian rhythm in EV production and function. In humans and rodents, the concentration of circulating EVs increases during the active phase and decreases over inactive phase 101-104. In mice, this was found to be inversely related to corticosterone levels 102. Murine lung-derived EVs were shown to have circadian rhythm related differences in uptake and functionality in an *in vitro* model using mouse-derived primary whole bone marrow cells 104. In contrast, milk-derived EVs from cows showed no difference in EVs or miRNA cargoes over the course of the day 105. This discrepancy may be due to the difference between milk and blood, where milk is stored in alveoli before being released to the cistern, allowing for EVs to be stored. Although *in vitro* studies represent a simplified model of the circadian rhythm, physiological changes including protein production, electrophysiology, and gene transcription have been reported to change over a 24-hour cycle in culture 106. EVs from cultured tendon fibroblasts showed changes in protein cargoes associated with RNA binding and actin-binding over the course of 24 hours 107. Together, these results suggest that EVs may communicate time-dependent information over the course of the circadian rhythm, and highlight the importance of collection time when investigating EVs as potential biomarkers.

**Figure 5:**
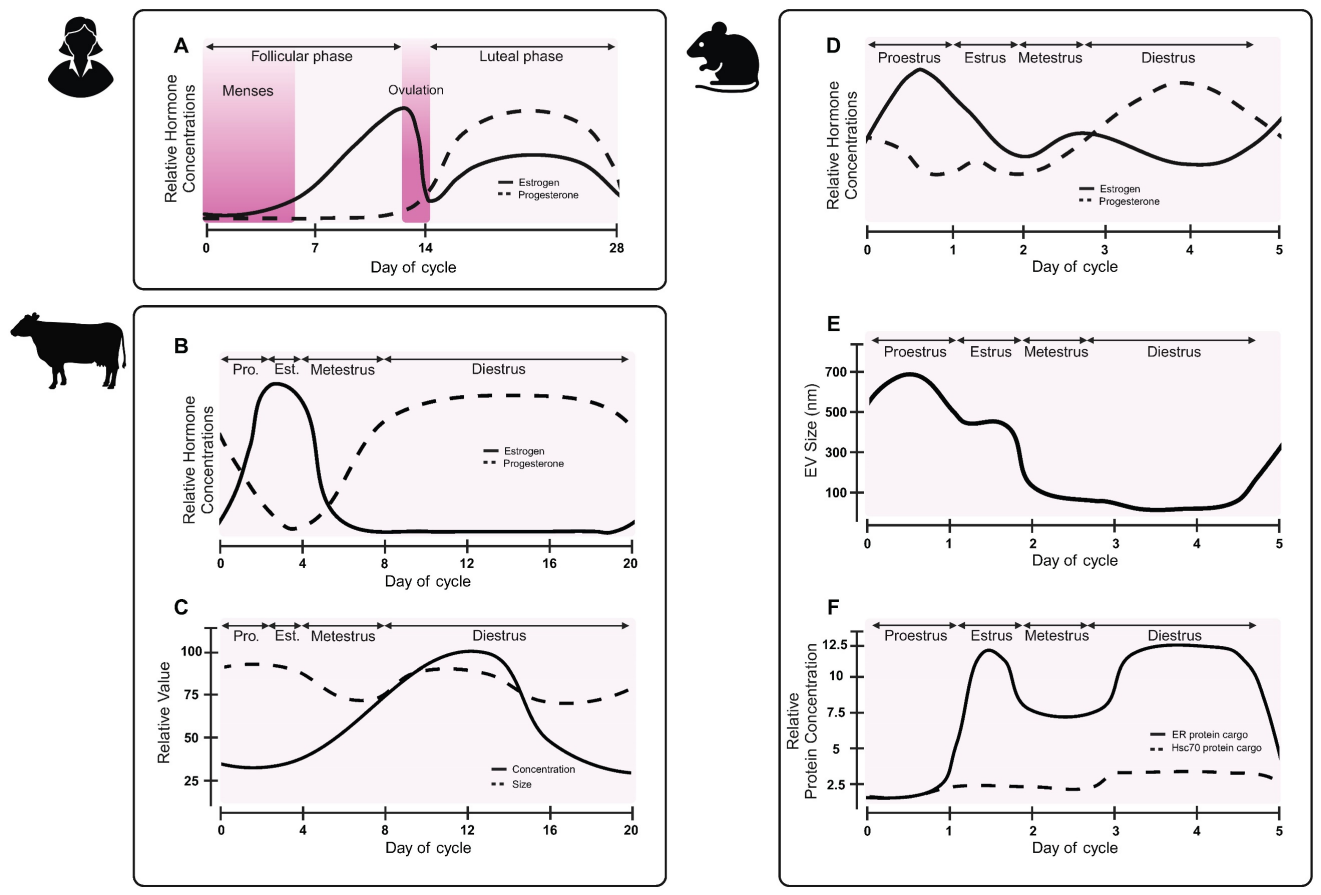
** EV characteristics vary over the reproductive hormone cycle.** While the effect of the human menstrual cycle on EVs has not been reported, preclinical studies in other species suggest that EV sizes, and potentially biogenesis, may change across the hormone cycle. (**A**) Hormone changes in the human menstrual cycle differ from hormone changes in the (**B**) bovine estrous cycle. (**C**) Concentration and size of bovine follicular fluid-derived EVs vary over the course of the estrous cycle. Adapted with permission from 111, copyright 2018 Springer Nature. (**D**) Hormones fluctuate during the mouse estrous cycle. (**E**) Murine luminal fluid-derived EV size changes over the course of the mouse estrous cycle. (**F**) Murine luminal fluid-derived EVs carry varying protein cargoes over the course of the estrous cycle. Adapted with permission from 112, copyright 2022 Springer Nature.

One under-investigated contribution to EV biogenesis is the menstrual cycle (**Figure 5A**). Changes in hormone levels have effects throughout the body, with observed differences in circulating proteins and tissue-specific mRNA expression 108, 109. In non-human placental mammals, females experience an estrous cycle. A bovine study revealed that follicular fluid-derived EVs carried variable miRNA cargoes over the course of the estrous cycle, with lowest concentrations of miRNA cargoes during the low progesterone phase 110. During high progesterone phases, EVs cargoes were enriched for miRNAs associated with immune cell regulation, whereas low progesterone phase EVs carried cargoes related to oocyte differentiation, gamete generation, and sexual reproduction. A separate study observed differences in size and concentration of EVs, as well as RNA cargoes over the bovine estrous cycle (**Figure 5B-C**) 111. A study on murine luminal fluid-derived EVs found an increase in size and protein cargoes in the high progesterone phase of the estrous cycle (**Figure 5D-F**) 112. Authors observed differences in estrogen receptor membrane proteins, suggesting that EVs may help to coordinate the estrous cycle and hormone signaling 112. While changes to EVs have not been explicitly studied over the human menstrual cycle, future work should consider how changes in hormones, either due to the menstrual cycle or hormonal contraceptives, may alter biomarker readouts.

In addition to biological variation, environmental and emotional stressors also play a role in the production and loading of EVs 113. One study evaluated circulating EVs in pregnant mice, revealing that stress decreased the concentration of circulating EVs compared to control animals 102. Food intake may also contribute to differences in EV concentrations and cargoes, with fasting individuals showing an increase in CD9 markers in isolated EVs 101, 102.

Finally, biological sex is an important consideration in biomarker identification, with reports of males having 10x more circulating EVs than non-pregnant females. After normalization to particle number, plasma-derived EVs from males contained 2x the total protein concentration as EVs from females 101. The work described here establishes the need for careful considerations when assessing EVs across a variety of biofluids. Time of day, food intake, hormone levels, and psychosocial stress all have a demonstrated effect on the production and composition of EVs. These variables should be considered in clinical studies to improve diagnostic potential of EVs and mRNA cargoes.

## 3. Extracellular vesicles as therapeutic carriers of mRNA

Whereas Section 2 focused on using EVs as biomarkers of disease, here we discuss the use of EVs as therapeutic carriers of mRNA for a spectrum of human health indications. The successful development of an mRNA vaccine for the SARS-CoV-2 virus (COVID-19) demonstrated the potential of mRNA-based vaccines and therapies to improve health outcomes. However, stability of mRNA in extracellular fluids presents a challenge to effective mRNA therapy. As a negatively charged macromolecule (approximately 1-15 kb), mRNA has difficulty crossing anionic cellular membranes and has a median intracellular half-life of approximately 7 hours. Furthermore, large amounts of mRNA remain trapped in endosomes after cellular entry and are unable to leak into the cytoplasm to enable translational functions to initiate protein production. While modifications can increase stability of mRNA in the cytoplasm to enable protein synthesis, the transport of mRNA into the cytoplasm of recipient cells requires safe and efficient delivery vehicles to provide mRNA with protection and facilitate its uptake as well as endosomal escape.

Biotechnology has enabled nanocarrier formulations for therapeutic mRNA delivery applications. mRNA can be efficiently delivered using synthetic lipid nanoparticles (LNPs), however, the modification of LNPs with coatings and ligands increases manufacturing complexity and costs, and may impact biocompatibility and biodistribution of the LNP formulations 114. EVs have emerged as a promising delivery platform given their innate targeting properties and lower immunogenicity compared to LNPs. A growing body of work demonstrates the benefits of using EVs as delivery vehicles and addresses the challenges associated with EV-based therapeutics, including scalable manufacturing and efficient loading. In this section, we discuss loading strategies, therapeutic applications of mRNA loaded EVs, and challenges to the translation of mRNA-loaded EVs (**Table 4**).

### 3.1 Engineering EVs as mRNA carriers

#### 3.1.1 Exogenous loading

**Figure 6:**
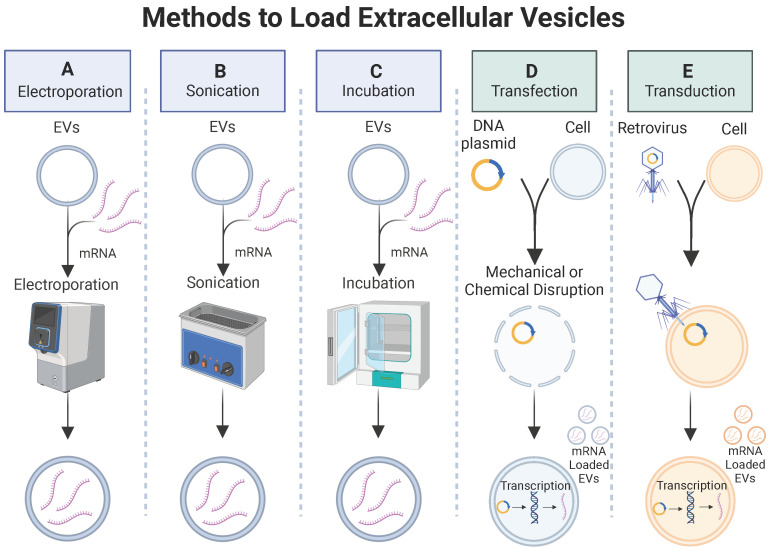
** Methods to load extracellular vesicles.** Exogenous methods such as (**A**) electroporation, (**B**) sonication, and (**C**) passive incubation and endogenous methods such as (**D**) transfection or (**E**) transduction can be used to load mRNA into EVs.

Exogenous loading involves passive or active (mechanical or chemical) methods to increase permeability of EV membranes, enabling the encapsulation of cargo molecules. Some exogenous loading techniques used to load small drug molecules and miRNAs into EVs include incubation, electroporation, sonication, and chemical membrane permeabilization (**Figure 6A-C**). Passive methods involve incubation of the EVs with cargo molecules while controlling parameters such as temperature and pH to promote cargo encapsulation. Mechanical methods, such as electroporation, apply an electrical field to create transient pores in an EV membrane, allowing cargo molecules to pass into the vesicle. Sonication uses sound energy to deform the EV membrane, enabling cargo encapsulation. Chemical membrane permeabilization relies on detergents (e.g., saponin) to create pores in the EV membrane allowing for the loading of cargo molecules into the EVs without destroying the membrane. Although loading of small drug molecules and miRNAs has been achieved via exogenous loading, there is little work demonstrating exogenous loading of large proteins or mRNA into EVs. To investigate the biodistribution of EVs delivered via inhalation to treat respiratory viruses, a recent study successfully used an established electroporation method to load green fluorescent protein (GFP)-encoding mRNA and red fluorescent protein (RFP) into lung-derived EVs 115. After demonstrating successful loading of GFP-encoding mRNA into EVs via electroporation, a follow-up study utilized electroporation to load COVID-19 spike (S)-protein encoding mRNA into EVs and LNPs as inhalable mRNA COVID-19 vaccines and observed significantly higher number of antibodies in mice treated with mRNA loaded EVs compared to LNPs 115. Both studies demonstrated successful loading of mRNA into EVs via electroporation and provide rationale to further investigate other exogenous methods for loading mRNA into EVs.

While exogenous loading of mRNA into EVs via electroporation is possible, the size and molecular weight of mRNA molecules may prohibit efficient mRNA loading into EVs. For example, when loading EVs with mRNA encoding proteins to activate prodrugs for human epidermal growth receptor 2-positive (HER2^+^) breast cancer, a separate group observed less mRNA in electroporated EVs than non-electroporated EVs, suggesting mRNA remained adhered to the surface of EVs and the failure of electroporation 116. Alternatively, transfected donors were shown to effectively load mRNA into EVs, demonstrating how challenges with mRNA loading via exogenous loading methods may be solved utilizing endogenous loading methods 116.

#### 3.1.2 Endogenous loading

Successful loading of mRNA into EVs has been demonstrated via endogenous loading 116-119. By genetically modifying cells to overexpress mRNA, the loading of desired mRNA cargoes in secreted EVs is enhanced. Genetic modification of the cells can be achieved through two common mechanisms: 1) transduction, which involves using a viral vector to introduce foreign DNA into a cell; 2) transfection, which utilizes mechanical or chemical methods to introduce DNA into a cell (**Figure 6D-E**). To investigate gene-delivered enzyme prodrug therapy (GDEPT) to treat HER2^+^ breast cancer, researchers used a retrovirus to deliver prodrug-activating protein encoded plasmids into MSCs, which then secreted EVs with prodrug-encoding mRNA for GDEPT therapy 117. While this study demonstrated successful production of mRNA encapsulated EVs via transduction, the use of viruses in gene delivery raise concerns with immune recognition, insertional mutagenesis, and inflammatory toxicity, demonstrating a need for alternative mRNA loading techniques to address safety concerns.

Transfection provides an alternative strategy for endogenous mRNA loading and avoids use of viral vectors for genetic modification, providing a safer gene delivery method. After observing low mRNA encapsulation in EVs after electroporation, researchers engineered a plasmid to transfect HEK-293 cells, resulting in the secretion of EVs enriched with prodrug-encoding mRNA to treat HER2^+^ breast cancer, improving upon electroporation loading strategies mentioned previously 116. In a separate study, a plasmid was engineered to transfect HEK-293 cells through mechanical manipulations. The secreted EVs encapsulated an mRNA coding for an anti-neuroinflammatory enzyme with proposed uses for the treatment of Parkinson's disease 118. These studies demonstrate effective endogenous loading of mRNA into EVs, providing viable strategies for loading other large therapeutic proteins and mRNA into EVs that cannot be achieved by exogenous loading methods.

Although transfection provides a safer technique for the endogenous loading of mRNA into EVs, the use of plasmids for the genetic modification of cells poses an inherent safety risk to patients. To further improve the safety of endogenous loading of mRNA into EVs through transfection, the use of plasmids was eliminated in one study by directly transfecting HEK-293 cells with mRNA, which was then packaged into secreted EVs 119. Higher EV loading efficiency was achieved by delivering mRNA to the cell cytosol in comparison to cells transfected with plasmids 119. By improving both safety and efficiency of loading methods for mRNA encapsulation, clinical translatability of EV therapeutics may become a reality.

### 3.2 EVs carry mRNA cargoes for cancer therapeutics

**Figure 7:**
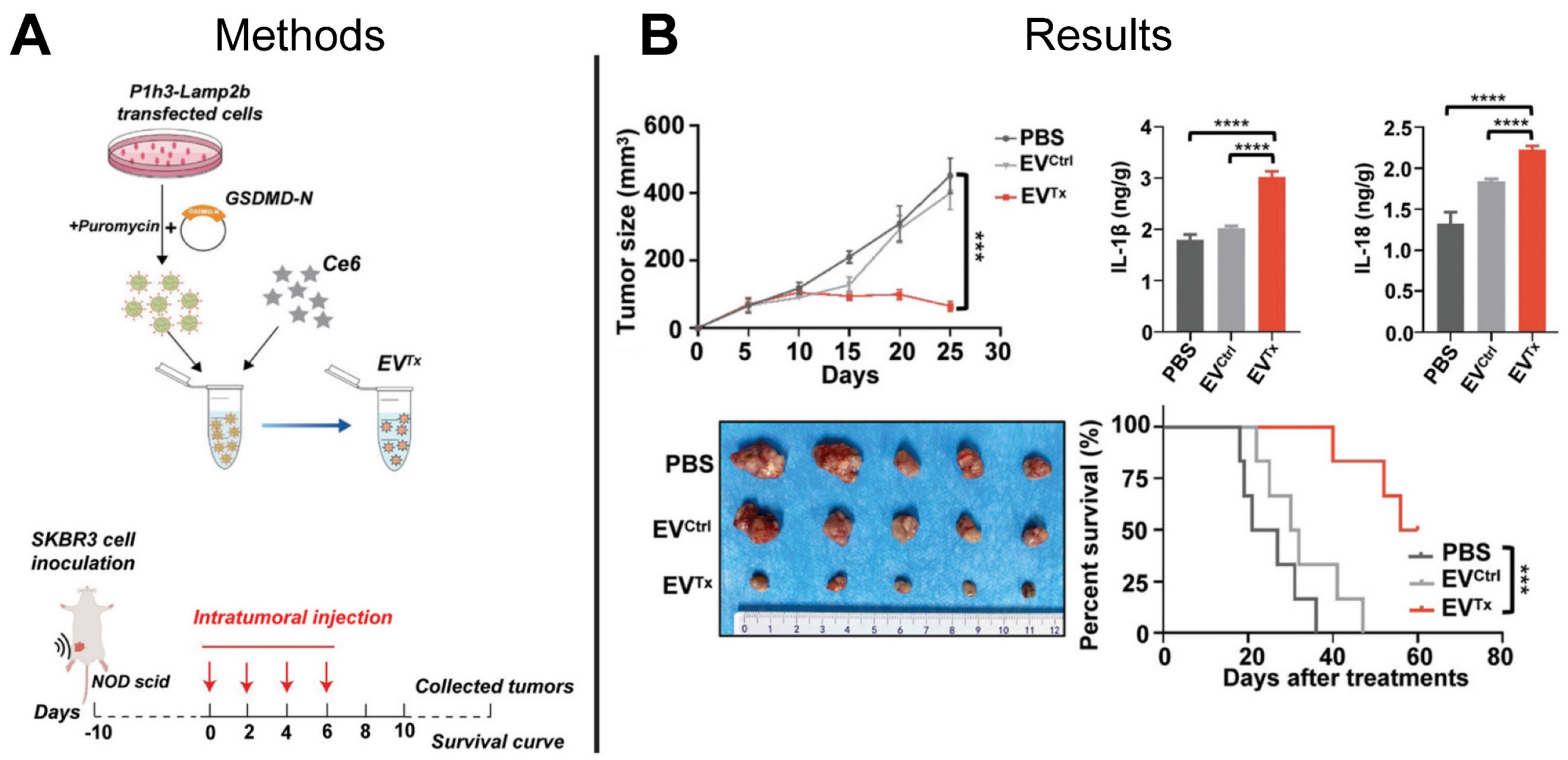
** EV-based mRNA gene therapy suppressed tumor growth in HER2^+^ mouse model.** (**A**) Prodrug therapy was mediated by EV delivery of GSDM-N mRNA and prodrugs to tumor cells. (**B**) *Ex vivo* imaging demonstrated suppression of tumor growth and increased survival of mice treated with mRNA-loaded EVs compared to control mice treated with PBS or empty EVs. Adapted with permission from 121, copyright 2023 John Wiley and Sons, Inc.

The Centers for Disease Control and Prevention reported more than 600,000 cancer-related deaths in 2021, with breast cancer being the leading cause of cancer-related death in women 120. Overexpression of the HER2 on the cell surface promotes cancer cell division and tumor growth and characterizes HER2^+^ breast cancer. Conventional chemotherapeutic approaches to treat HER2^+^ breast cancer lack tumor selectivity, contributing to insufficient drug concentrations in tumors and development of drug resistance. Immunotherapy provides an alternative to conventional HER2^+^ breast cancer chemotherapeutics by inducing immunogenic cell death pathways such as necrosis, apoptosis, or pyroptosis. Pyroptosis is inflammatory programmed necrosis characterized by swelling and rupture of cells, induced by the overexpression of the protein gasdermin (GSDMD) in the cellular membrane, suggesting direct delivery of GSDMD to cancer cells could be an effective cancer treatment. Although an effective technique to deliver GSDMD protein to cancer cells presents a challenge, EV-mediated delivery of functional GSDMD mRNA to cancer cells offers a promising solution. EVs expressing anti-HER2^+^ antibodies were used in a recent study to deliver GSDMD mRNA to HER2^+^ cancer cells in a HER2 overexpressing mouse model resulting in induced pyroptosis and a tumor immune response that significantly suppressed tumor growth and prolonged mouse survival compared to control mice that were treated with either PBS or unloaded EVs (**Figure 7A-B**) 121. This study demonstrated the feasibility of using EVs to deliver exogenous mRNA to tumors to induce tumor immune response to treat HER2^+^ breast cancer, setting the stage for engineered EVs to be used for other cancer immunotherapy targets.

GDEPT is an alternative to traditional immunotherapy, where a prodrug inert to native human enzymes and harmless to human cells and tissues, transforms into a cytotoxic drug upon activation by an enzyme encoded by viral or bacterial genes. However, GDEPT requires direct gene injection at the cancer site for treatment to be effective, which may be achieved through EV-mediated delivery of exogenous mRNA to the cancer cells. In one such example, mRNA-loaded EVs expressing anti-HER2^+^ antibodies were systemically administered to deliver functional HchrR6 mRNA with the prodrug C_16_H_7_CIN_2_O_4_ (CNOB) to HER2^+^ cancer cells in a HER2 overexpressing mouse model and achieved significant near-complete growth-arrest of tumors compared to controls treated with free prodrug or unloaded EVs 116. However, CNOB is a new prodrug and has not undergone clinical testing to establish a safe dose, presenting a barrier to the clinical translation of CNOB for GDEPT to treat HER2^+^ breast cancer. To improve clinical prospects of GDEPT with HchrR6 mRNA for HER2^+^ breast cancer treatment, a follow-up study administered EVs both loaded with functional HchrR6 mRNA and labeled with anti-HER2^+^ antibodies with tretazicar, a clinically tested prodrug with a well-established safe dose, in addition to CNOB, and observed growth-arrest of HER2^+^ human breast cancer tumors 119. While both studies achieved near-complete growth-arrest of HER2^+^ breast cancer tumors *in vivo*, neither study achieved tumor regression or elimination to convey a complete cancer treatment. Future work could investigate utilizing GDEPT in combination with other cancer treatment methods that achieve both tumor regression and elimination to demonstrate a complete cancer treatment.

GDEPT utilizing a suicide gene to activate a prodrug is a promising treatment method for glioma, a common brain tumor that originates within the glial cells that support the neurons of the brain. Glioma can be extremely difficult to treat due to its tendency to infiltrate neighboring brain tissue. Studies have conveyed the therapeutic potential of mesenchymal stem cells (MSCs) expressing the yeast cytosine deaminase::uracil phosphoribosyl transferase (yCD::UPRT) suicide gene, which converts the prodrug 5-fluorocytosine (5-FC) to 5-fluorouracil (5-FU) to induce cell death. Whereas MSCs are rapidly cleared by the liver (<24 h post-injection), EVs derived from MSCs and loaded with mRNA showed increased retention, which may improve tumor cell death 117. Based on this rationale, researchers administered mRNA loaded EVs to deliver yCD::UPRT mRNA with the prodrug 5-FC to glioma tumor cells in an *in vitro* glioma cell model and triggered significant tumor cell death as compared to controls not treated with the 5-FC prodrug 117. Although preclinical studies have demonstrated suicide gene delivery via engineered MSCs, additional *in vivo* animal studies are needed to evaluate suicide gene delivery by MSC derived EVs.

Glioma can be attributed to loss of function of the phosphatase and tensin homolog (PTEN) gene, which codes for the PTEN enzyme that regulates cell division and acts as a tumor suppressor, suggesting the direct delivery of PTEN to tumor cells could be an effective treatment for glioma. However, an effective method to deliver PTEN protein to tumor cells has not been established, but the EV-mediated delivery of functional PTEN mRNA to cancer cells provides a viable solution. A recent study utilized loaded EVs to deliver PTEN mRNA to PTEN-deficient glioma tumor cells in a glioma mouse model and demonstrated tumor growth inhibition and increased animal survival in PTEN-deficient mice as compared to control animals treated with PBS or control EVs 122. For clinical translation of EVs, the optimal cell source to derive the EVs needs to be identified, which may require evaluation of multiple allogenic and autologous cell sources.

Melanoma is a common form of skin cancer attributed to genetic mutations with melanocytes, cells that produce melanin, causing cells to grow and divide uncontrollably into malignant tumors. Therapeutic mRNA vaccines have emerged as a promising method to treat melanoma tumors, using functional mRNA for antigens that are expressed on the surface of antigen presenting cells, which directly interact with T cells to induce a robust tumor-specific T cell immune response. However, delivery of mRNA is challenging due to its large molecular weight and negative charge, which may be overcome utilizing extracellular vesicles to deliver mRNA for therapeutic vaccines. Researchers in one study decorated outer membrane vesicles (OMVs) with mRNA to deliver ovalbumin mRNA to melanoma tumor cells in a melanoma mouse model and demonstrated significant inhibition of melanoma tumor progression than control animals treated with LNPs 123. This study demonstrated a mRNA-based vaccine technology delivered by EVs that could be applied to other types of cancer.

### 3.3 EVs deliver mRNA for the treatment of infectious diseases

**Figure 8:**
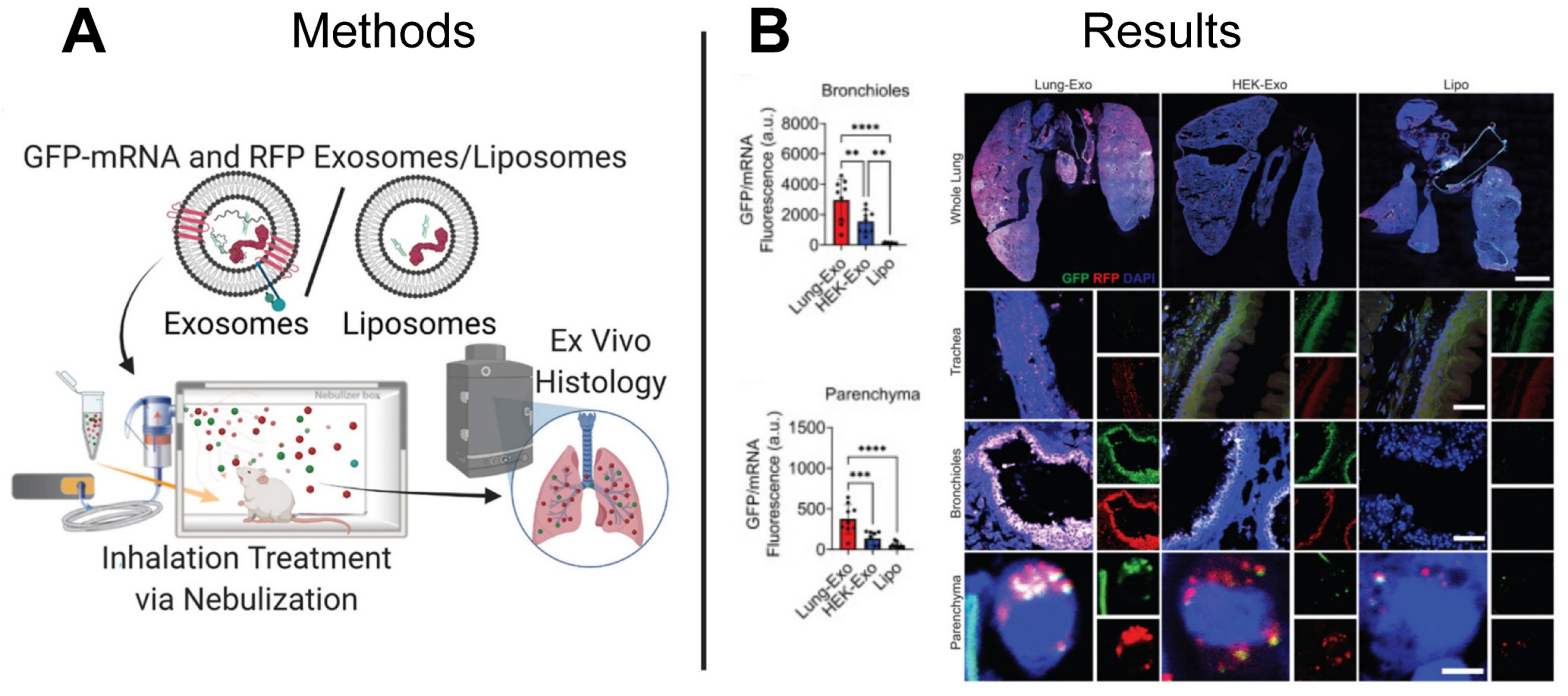
** EV-based mRNA delivery enhances pulmonary bioavailability in mouse model.** (**A**) Lung-derived EVs (Lung-Exo), commercially available EVs (HEK-Exo), and liposomes (Lipo) were used to deliver GFP-mRNA and RFP to mice through inhalation treatment via nebulization. (**B**) Greater GFP protein expression in the bronchioles and parenchyma regions of the lung in mice treated with Lung-Exo EVs compared to control mice treated with HEK-Exo EVs or LNPs suggests Lung-Exo EVs enhanced pulmonary bioavailability, retention, and therapeutic efficacy. Adapted with permission from 125, copyright 2022 Elsevier.

In addition to cancer, EV-based therapeutics have been proposed to treat infectious diseases. Spread by viruses and bacteria, infectious diseases are the third main cause of death in the US, with COVID-19 contributing to more than 400,000 deaths in 2021 124. Although mRNA-based LNP vaccines developed by Pfizer-BioNTech, Moderna, and Janssen are authorized by the U.S. Food and Drug Administration for the immunization against COVID-19, their intramuscular delivery limits pulmonary bioavailability and can cause off-target side effects. Inhaled therapeutics delivered directly to the airway provide an alternative to intramuscular injection and can improve pulmonary bioavailability and minimize adverse off-target side effects. While LNP vaccines have demonstrated efficacy against COVID-19 as intramuscular injections, inhaled LNPs require reformulation to improve biocompatibility with the lung microenvironment. In contrast, EVs derived from cells in the lung naturally express membrane features recognized and favored by the lung microenvironment and may already be optimized for mRNA delivery. Although lung-derived EVs may effectively evade immune clearance and target pulmonary cells, the distribution and retention of inhaled lung-derived EV therapeutics is not yet well understood. To elucidate the biodistribution and retention of therapeutic EVs delivered through nebulization, a research group delivered green fluorescent protein (GFP) mRNA loaded EVs to the lungs of mice and observed a 24.1-fold and 22.9-fold increase in protein expression in the bronchioles and parenchyma lung regions than control mice treated with GFP mRNA loaded LNPs (**Figure 8A-B**) 115. Demonstrating superiority of EVs to LNPs, this study provides motivation for development of inhaled EV-based mRNA vaccines.

The mRNA LNP-based vaccines deliver COVID-19 S-protein mRNA to activate an immune response to elicit antibody protection. After demonstrating successful mRNA delivery to pulmonary cells in lungs via lung-derived EVs, the same group utilized nebulization to deliver S-protein mRNA-loaded EVs to the respiratory tract of mice and demonstrated a significantly greater production of immunoglobin G (IgG) and secretory IgA (SIgA) compared to control mice dosed with a comparable LNP formulation 125. Furthermore, when vaccinated mice were exposed to wild-type COVID-19 pseudoviral particles, mice vaccinated via EVs showed significantly greater viral clearance compared to mice vaccinated via LNPs, suggesting EVs offer more rapid protection against viral infection 125. Taken together, these results highlight the efficacy of EVs as inhaled, or locally delivered, therapeutics and vaccines.

mRNA-based therapeutics also provide an effective treatment strategy for infectious diseases. With approximately 1.2 million Americans diagnosed with HIV-1 infections, and over 30,000 new cases each year, there is an unmet need for new therapies to treat HIV-1 126. The current approach to managing HIV-1 infection involves combination antiretroviral therapy (cART), but cART is associated with life-shortening side effects and the development drug resistance. Alternative strategies use long-term epigenetic repression of viral expression using DNA methyl transferase 3A (DNMT3A) which down-regulates HIV-1 transcription via DNA methylation. DNMT3A fused to the promoter-targeting Zinc Finger Protein (ZFP-362) can enrich methylation, but delivery to multi-tissue HIV-1 reservoirs for long-term virus inactivation is a challenge. After engineering a DNMT3A and ZFP-362 construct (ZPAMt), researchers administered ZPAMt mRNA loaded EVs to immunodeficient mice infected with HIV-1 and observed a significant reduction in viral load compared to control mice treated with cART 127. This study opens the door for future investigations into using EVs to deliver epigenetic modulators for the treatment of infectious diseases.

### 3.4 EVs prove useful as therapeutic carriers for neurological diseases

**Figure 9:**
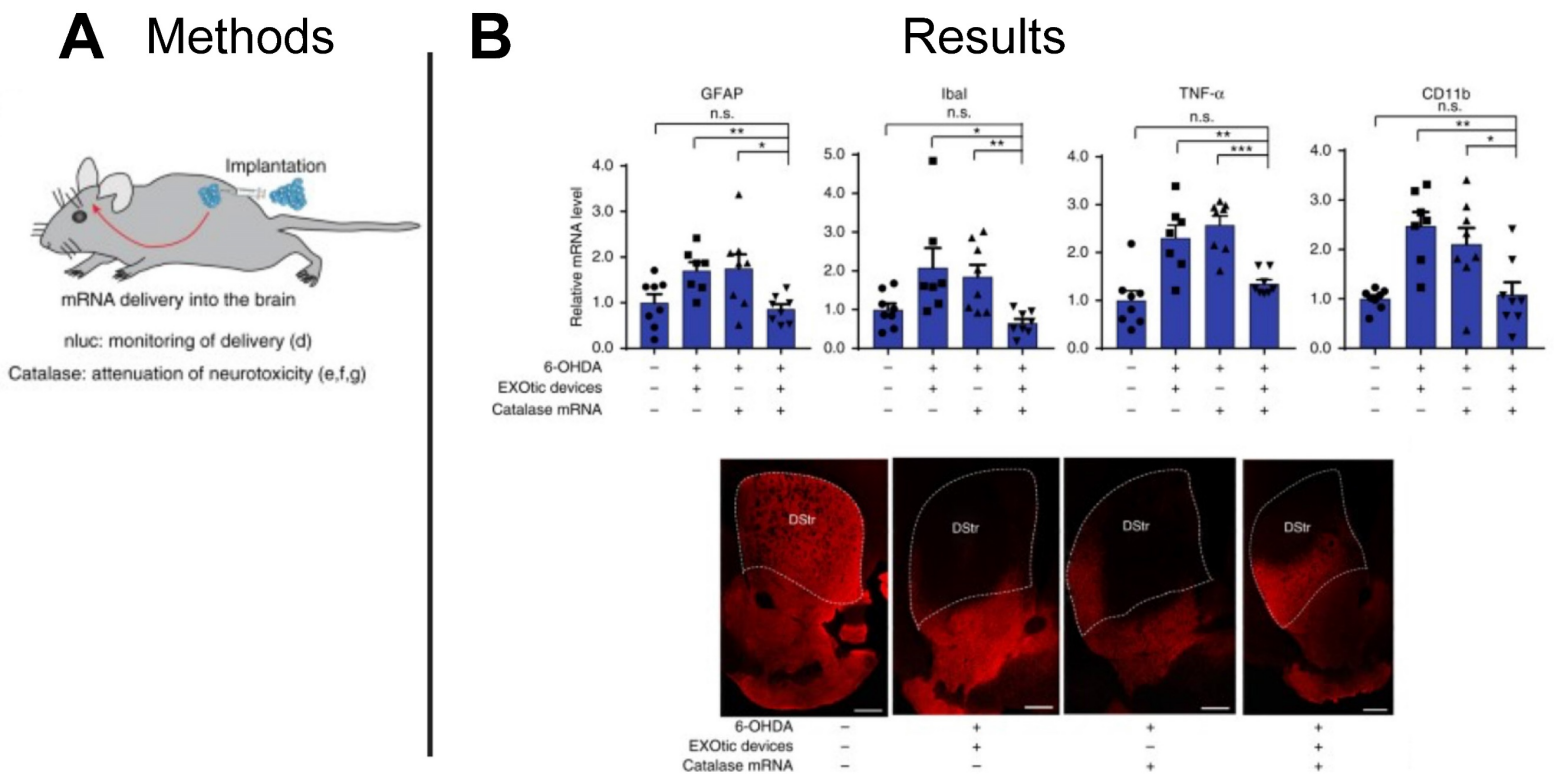
** EV-delivered mRNA attenuated neuroinflammation in Parkinson's disease model.** (**A**) EVs were used to deliver catalase mRNA to attenuate neurotoxicity and neuroinflammation. (**B**) Decrease in the expression of neuroinflammation markers (GFAP, Iba1, TNFα, CD11b), and immunostaining for TH^+^ neurons suggest EV-delivered mRNA reduced neuroinflammation. Adapted with permission from 118, copyright 2018 Springer Nature.

EVs have also been proposed as therapeutic carriers for treating neurological disease. With increases in life expectancy, the US has seen an increase in neurodegenerative disorders, with Parkinson's disease being one of the most common 128. Catalase is known to attenuate neuroinflammation and to reduce Parkinson's disease symptoms, however, the blood brain barrier (BBB) presents a challenge to the effective delivery of catalase. With the ability to carry nucleic acid cargoes across biological barriers for cellular communication, EVs present an ideal vehicle to deliver functional catalase mRNA to target cells in the brain tissue. In a mouse model of Parkinson's disease, researchers delivered catalase mRNA loaded EVs to target cells in the brain and demonstrated significantly lower expression of neuroinflammatory markers (GFAP, Iba1, TNFα, CD11b) compared to controls dosed with free catalase mRNA, establishing an EV-based mRNA delivery platform for treating Parkinson's disease (**Figure 9A-B**) 118. Given inherent barrier crossing capabilities of EVs, other neurological indications may benefit from EV-based mRNA therapeutics capable of penetrating the BBB.

### 3.5 EVs can carry mRNA for the treatment of cardiovascular disease

Inflammation attenuating mRNAs are useful in the treatment of cardiovascular disease. Cardiovascular disease is a leading cause of death in the U.S. with 700,000 deaths in 2021 129. Atherosclerosis is a chronic inflammatory condition characterized by plaque buildup in artery walls that contributes to the progression of coronary artery disease and other cardiovascular diseases. Current clinical trials are exploring treatment strategies for atherosclerosis including the use of anti-inflammatory therapies, such as the IL-1β inhibitor Canakinumab, or therapies that interfere with inflammatory signaling pathways, such as the p38MAPK inhibitor Losmmapimod. However, these therapeutics require frequent injection and have side-effects such as liver-toxicity. IL-10, a soluble cytokine produced by macrophages and regulatory T cells, provides a potential alternative to conventional anti-inflammatory treatments for atherosclerosis, although effective delivery of IL-10 that avoids off-target effects remains a significant challenge. Using a mouse model of atherosclerosis, researchers delivered functional IL-10 mRNA encapsulated in EVs to macrophages, demonstrating a 10% reduction in the size of atherosclerotic plaques as compared to control animals treated with PBS or empty EVs 130. This study demonstrated EV-based delivery of IL-10 mRNA for treatment of atherosclerosis, providing a foundation for developing other EV-based mRNA delivery platforms to treat inflammatory diseases.

**Figure 10:**
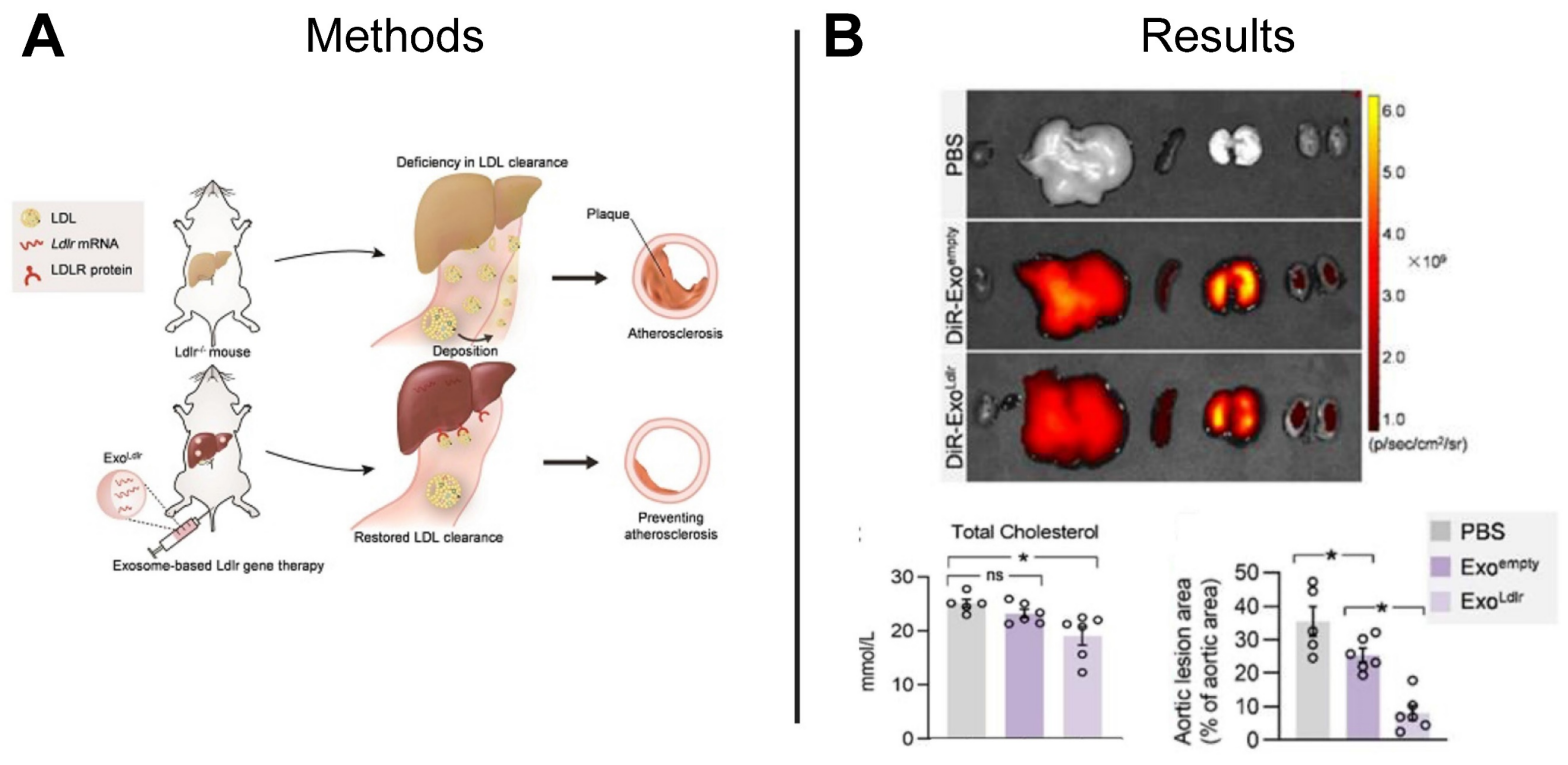
** EV-based mRNA restored LDLR function in hypercholesterolemia mouse model.** (**A**) EV-based mRNA was used to restore LDLR function in a hypercholesterolemia mouse model. (**B**) *Ex vivo* imaging demonstrated distribution of EVs to various organs following tail-vein injection, and significant reductions in total cholesterol and aortic lesion area was observed in mice treated with *Ldlr*-loaded EVs compared to mice treated with empty EVs or PBS. Adapted with permission from 133, copyright 2021 Ivyspring International Publisher.

The development of atherosclerotic plaques can be attributed to familial hypercholesterolemia, an inherited genetic disease characterized by high low-density lipoprotein (LDL) cholesterol levels caused by a functional loss mutation of the low-density lipoprotein receptor (Ldlr) gene 131. Studies suggest CRISPR/Cas gene editing technology may restore function to the Ldlr gene, however, the gene-editing efficiency of Cas9 is low and functionality of the cells is unknown 132. The repeated delivery of functional mRNA provides an alternative to a one-time gene correction and can be achieved using mRNA-loaded EVs. Compared to liposome and viruses that are commonly used as nanocarriers for gene therapy, EVs can be derived from patient-specific cells and result in less cytotoxicity and immunogenicity. Recently, researchers delivered Ldlr mRNA encapsulated in EVs to hepatocytes, restoring Ldlr function in a transgenic mouse model of hypercholesterolemia and significantly lowering serum LDL-cholesterol as compared to control animals treated with PBS or empty EVs (**Figure 10A-B**) 133. Together, these results establish a foundation for using EV-mediated mRNA delivery to treat inherited diseases.

While EV delivery of Ldlr mRNA demonstrated the effective treatment of hypercholesterolemia, the loading inefficiency of EV-based mRNA delivery remains a significant barrier to translation. Recent studies suggest RNA-binding proteins (RBPs) that interact with the therapeutic mRNA can be engineered to fuse to EV membrane proteins to facilitate encapsulation of target mRNA. Recently, researchers used an RNA aptamer with a base-pair sequence that matched Ldlr mRNA and recognized by a bacteriophage coat protein (MCP) fused to an EV membrane protein (CD9). Higher levels of Ldlr mRNA in EVs was achieved using the RNA aptamer, bacteriophage coat protein, and EV membrane protein complex to load mRNA compared to controls that did not 134. Furthermore, the authors administered EVs carrying LDLR mRNA to hepatocytes to restore LDLR function in a transgenic mouse model of hypercholesterolemia and demonstrated significantly lower serum LDL cholesterol levels as compared to control animals dosed with PBS 134. Although mRNA loading efficiency presents a significant challenge to EV-based mRNA delivery, improved loading of mRNA using RBPs may accelerate the translation of EV therapeutics.

Atherosclerosis can impair blood flow to the brain causing acute bran injury and ischemic stroke, the leading neurological cause of disability and death with over 77 million cases reported in 2019 135. With protective properties that reduce apoptotic cell death and infarct volume, ameliorate delayed neuronal death, and promote cell survival and neural regeneration, nerve growth factor (NGF) has emerged as a promising therapeutic for the treatment of acute cerebral ischemia symptoms. Acute cerebral ischemia is characterized by the decrease of endogenous expression of NGF, demonstrating the need to deliver exogenous NGF to the infarcted cortex to aid in recovery. However, systemically administered NGF is subjected to both fast enzymatic degradation and poor permeability of the blood brain barrier (BBB), making effective delivery of NGF challenging. With the ability to transverse biological barriers while carrying cargoes including nucleic acids, extracellular vesicles provide the ideal platform to deliver NGF to treat acute cerebral ischemia. In a recent study, NGF mRNA loaded EVs were delivered to the ischemic cortex in a mouse model of cerebral ischemia and demonstrated a significant reduction in classically activated microglia, associated with inflammation and an increase in alternatively activated microglia, associated with restorative processes as compared to controls animals dosed with either saline or empty EVs 136. Future work may take inspiration from this study to develop mRNA delivery platforms for a range of neurological diseases.

### 3.6 Challenges associated with engineering EV therapeutics to deliver mRNA

In the previous sections, we briefly discuss challenges to EV-based mRNA delivery therapies, such as development of methods for the effective loading of therapeutic mRNA cargo into EVs. While effective loading of siRNA cargo into EVs has been demonstrated via electroporation, effective loading of higher molecular weight mRNA cargo into EVs has not been achieved 137. Endogenous mRNA loading has proven effective, such as the transfection of HEK293 cells to produce EVs containing mRNAs for cancer and neurological therapeutic applications, however EV-based therapeutics often carry a low number of cargo molecules (one intact long RNA molecule per 1,000 EVs 1) so improvements in loading and scalable manufacturing are needed for translation of EV-based therapeutics 116, 118. Here we discuss challenges to and considerations for engineering EV therapeutics, including scalable manufacturing (upstream and downstream), unloading of endogenous cargo, efficient loading approaches, and targeted drug delivery.

Scalable manufacturing presents many challenges to translation of EV-based mRNA therapies. First, an appropriate parent cell must be identified to support the intended therapeutic application. Recently, researchers developing a vaccine for COVID-19 compared the biodistribution of EVs from HEK cells (HEK-EV) to EVs from lung cells (Lung-EV) for mRNA delivery and achieved a 1.3-fold increase in mRNA biodistribution in lungs via Lung-EV compared to HEX-EV, indicating the specific tissue targeting that exists for certain EVs 115, 125. Another hurdle to the translation of EVs is production of EVs in sufficient quantities for clinically relevant lot sizes. Bioreactor culture systems offer more cell growth area which can increase EV production. Within a bioreactor, dynamic conditions in which media is replenished and EVs are harvested from conditioned media yield increased EV quantities 138. Furthermore, physical, mechanical, or chemical manipulations may enhance the release of EVs from cells. Stressors such as shifts in temperature, pH, oxygen saturation, and nutrient availability alter EV production, however these changes in production may be cell type-specific, so must be evaluated as such 139-142. Mechanical perturbations such as nanoporation or electroporation also may alter EV production, with a recent study demonstrating a 10-fold increase in EVs produced per cell using cellular nanoporation compared to bulk electroporation 143.

Following EV production, development of scalable EV isolation processes remains a challenge. Methods to isolate and purify EVs involve a combination of differential centrifugation and filtration, however, it can be challenging to scale-up these processes to support current good manufacturing practices 144. Recent work demonstrated a significantly higher yield of isolated EVs utilizing ultrafiltration followed by liquid chromatography compared to differential ultracentrifugation 145. Authors demonstrated scalability of ultrafiltration and liquid chromatography EV isolation processes, suggesting viable options to support clinical translation of EV-based therapeutics 145. Beyond isolation of EVs, the long-term storage and stability of EVs presents another challenge. Recent studies suggest EVs should be stored at -80^o^C to maintain their physical characteristics and identified PBS containing human albumin and trehalose as an optimal storage buffer 146. However, these optimal storage conditions present a challenge to using EV-based therapeutics for mRNA delivery in resource-poor settings where deep-freezing storage may not be accessible. To address this challenge, researchers manufactured a lyophilized EV-based mRNA vaccine and demonstrated stability of the EV-based vaccine in ambient conditions for up to 28 days 125. Scalable manufacturing strategies will enable translation of EVs for mRNA delivery applications.

The endogenous cargoes of some manufactured EVs may pose safety risks and require unloading. For example, cargo unloading may be required for EVs derived from tumor cells. While tumor-derived EVs are ideal for delivering chemotherapeutic agents due to their inherent tumor targeting ability, facilitating the delivery and distribution of chemotherapeutics throughout specific tumor tissues, tumor-derived EVs are associated with cancer progression and may promote tumor growth via delivery of endogenous cargoes. By permeabilizing the membranes of tumor-derived EVs using saponin, a recent study demonstrated reduced protein and RNA cargoes, compared to untreated EVs 147. Importantly, saponin treated tumor-derived EVs retained their surface proteins and tumor targeting ability, demonstrating the feasibility of removing dangerous endogenous cargo from EVs without comprising physical characteristics that make EVs ideal for drug delivery.

Targeted drug delivery is a challenge, even with EVs that possess inherent targeting capabilities. In a recent study, researchers transfected cells to produce EVs with high affinity anti-HER2 antibodies to target the HER2 receptors in HER2 overexpressing cancer cells and demonstrated a greater uptake of EVs expressing antibodies compared to EVs not expressing antibodies 116. In a follow up study, the same group evaluated the therapeutic potential of EVs expressing anti-HER2 antibodies in a HER2 overexpressing mouse model and observed a 50% more pronounced arrest of xenograft growth compared to control EVs not expressing HER2 antibodies 119. Although these studies engineered EVs for targeting HER2 overexpressing breast cancer cells, this technology could be modified to other targeting ligands to create EVs for delivering mRNA to any disease that is characterized by the overexpression of receptors such as PSMA (prostate cancer), bombasin (gastric cancer), folate (epithelial cancer), and transferrin (breast cancer). In a recent glioblastoma study, researchers modified the surface of IFN-γ mRNA loaded EVs with anti-CD71 and anti-programmed cell death-ligand 1 (PD-L1) antibodies and demonstrated an increase in uptake and antitumor activity as compared to unmodified EVs 148. In another study, researchers investigating pancreatic cancer modified the surface of TP53 mRNA loaded EVs with a tissue targeting peptide and a humanized monoclonal antibody and demonstrated suppressed tumor growth and extended animal survival compared to LNPs 149. The continued development of techniques to modify EVs for enhanced targeting will further the translation of EV-based therapeutics for a range of diseases.

The release of mRNA into the cytosol of recipient cells initiates translation and protein expression. While mRNA encapsulated in EVs may be protected from enzymatic degradation by RNases, functional mRNA often remains trapped within the EVs where it eventually becomes degraded 1. This presents a challenge to translation of EV-based mRNA delivery for therapeutic applications. mRNA can be released via endosomal escape as the EVs fuse to the cell's plasma membrane. To facilitate endosomal escape, researchers engineered a novel EV containing a “releaser” and simultaneously delivered it to recipient cells with EVs containing functional mRNA cargo and achieved a 3-fold increase in protein expression, suggesting a viable mRNA release method 123. Alternatively, manipulating the porosity of the EV membrane may also enable endosomal escape. To initiate endosomal escape, researchers engineered an EV to express a pore-forming toxin, which stimulated the formation of pores on endosomal membranes to facilitate mRNA release, and demonstrated a significant increase in protein expression compared to control EVs 134. mRNA molecules are chemically unstable and may degrade after release into the cytosol, so a recent focus on mRNA design has revealed opportunities to improve stability, prolong half-life, and avoid off-target effects. Recently, researchers using LNPs for COVID-19 vaccination created an algorithm to improve the structure and codon usage of S-protein mRNA to improve stability and protein expression 150. Authors achieved 128x the antibody response than conventional codon-optimized benchmark, demonstrating the usefulness in tuning mRNA design to enhance therapeutic efficacy 150. Innovative strategies that overcome challenges to mRNA cargo release and mRNA stability will facilitate translation of EV-based mRNA delivery therapeutics.

## 4. Conclusion

There is tremendous potential in using EVs for the detection and delivery of mRNA cargoes. EVs reflect the state of their parent cell, allowing for insight into tissue function related to disease onset, disease progression, and response to treatment. Recent studies show promising results in using EVs for biomarkers of cancer, infectious diseases, neurological disorders, and women's health conditions. However, for effective translation of these biomarkers, procedural and biological variables must be considered. In addition to their use as biomarkers of disease, EV technologies hold great promise as mRNA-based therapeutics to improve human health outcomes. EVs help to overcome issues with the stability of mRNA *in vivo*, and recent advances demonstrate efficient methods of loading EV therapies with mRNA cargoes. While there are challenges to the translation of EVs as therapeutic delivery platforms for mRNA, such as manufacturing and loading, these challenges also provide great opportunities for future work to contribute to the development and translation of EVs as mRNA therapeutic carriers.

## Figures and Tables

**Table 1:** ** Diagnostic applications of EVs and non-mRNA cargoes.** Disease biomarker changes reported with respect to controls.

Indication		EV Source	Isolation Method	Size	Biomarker	Source
Cancer	Bladder Cancer	Human Urine	Ultracentrifugation	<200 nm	↑ HSP90 ↑ SDC1 ↑ MARCKSL ↑ TJP2 ↑ CD55	47
Infectious diseases	COVID-19	Human Serum	Immuno-precipitation (CD9, CD63)	100-150 nm	↑ COPB2	48
	HIV-1	Human Plasma	Precipitation	~250-350 nm	↑ EV concentrations (52.31 ± 5.72 OD 450 nm/min) ↑ EV size (1017 ± 159.5 nm) ↑ DAP-3	52
	Tuberculosis	Human Serum	Precipitation	-	*M. tuberculosis* peptides as EV cargo	56
Neurological Diseases	Alzheimer's Disease	Homogenized Mouse Brain Tissue	Ultracentrifugation	~100-150 nm	↑ Psen1 ↑ App ↑ Itgax ↓ Wdr61 ↓Pmpca ↓ Aldh1a2 ↓ Calu ↓ Amp32b ↓ Actn4 ↓ Ndufv2	64
	Parkinson's Disease associated cognitive decline	Human Plasma	Membrane affinity spin columns	~30-120 nm	↑ EV-associated tau ↑ EV- associated Aβ1-42	65
	Parkinson's Disease	Human Serum	Ultracentrifugation	~100 nm	hsa-miR-19b-3p hsa-miR-374b-5p hsa-miR-9-5p hsa-miR-374a-5p	66
	Autism Spectrum Disorder	Human Serum	Membrane affinity spin columns	~100 nm	↑ EV concentration ↑ Total EV protein concentration	72
	Depression	Human Saliva	Ultracentrifugation	~50 nm	↑ M6a	79
Obstetric Health	Preeclampsia	Human Plasma	Immuno-magnetic pulldown (CTB)	-	↑ CD105 ↑ IL-6 ↑ Placental growth factor ↑ Tissue inhibitor of metallopeptidase 1 ↑ Atrial natriuretic peptide	82
	Preeclampsia	Human Plasma	Size exclusion chromatography	~100-300 nm	(In second trimester) ↑ CD63 ↑IL-21 ↑ IL-22, ↑ TGF-β	83
	Gestational diabetes	Human Gingival Crevicular Fluid	Precipitation	148 ± 57 nm	↑ EV concentration	151
	Pregnancy loss	Human Plasma	EV Array	-	↓ CD9	84
	Preterm Birth	Human Plasma	Ultracentrifugation	30-120 nm	hsa-let-7a-2-5p hsa-miR-6759-5p hsa-miR-517b-5p hsa-miR-550a-3-5p	86
	Prenatal chromosomal testing	Human Maternal Plasma	Precipitation	30-90 nm	↓ Cell-free DNA concentration	87

**Table 2:** ** Diagnostic applications of mRNA.** Disease biomarker changes reported with respect to controls.

Indication		Biological Matrix	mRNA Biomarker	Source
Cancer	Colorectal Cancer	Human Serum	↑ Metadherin	44
Neurological diseases	Parkinson's Disease	Human Blood	↑ IFIT1 ↑ IFIT2 ↑ IFIT3 ↑ IFI6 ↑ IRF7 ↓POLR3B ↓ CAMK4 ↓ OWAR6 ↓ LINC00861 ↓ AC083843.1	67
	Parkinson's Disease	MPTP-induced PD mouse peripheral blood	↓ Vcp	68
		MPTP-induced PD mouse primary heterogenous brain tissues		
		Human Peripheral Blood		
	Autism Spectrum Disorder	Human Peripheral Blood	↓ SOD2 ↓ ERRα ↓ RORA ↓ GPER	74
	Autism Spectrum Disorder	Human Peripheral Blood	↓ OPRM1 ↓ PRKG1 ↓ HTR1E ↑ SCN9A ↑ DRD4 ↑ OPRL1 ↑ TACR1	76
Obstetric indications	Low Fertility	Primary Bovine Ovarian tissue	(Luteal phase) ↑ 161 mRNAs ↓ 296 mRNAs	152
			(Follicular phase) ↑ 253 mRNAs ↓ 222 mRNAs	

**Table 3:** ** Diagnostic applications of EV mRNA cargoes.** Disease biomarker changes reported with respect to controls.

Indication		EV Source	EV Isolation Method	EV Size	Biomarker	Source
Cancer	Colorectal Cancer	Culture media of Colorectal cancer cell lines (SW620, Wi-Dr, LS174T, HCT116) Normal colon fibroblast (CCD-18Co)	Ultracentrifugation	-	↑ VEGF ↑ CD133	43
		Human Plasma	Immuno-magnetic bead pull-down (CD9, CD63, and CD81)			
	Prostate Cancer	Human Plasma	Precipitation Ultracentrifugation Iodixanol velocity gradient Immunocapture	~100 nm	↑ AR-V7 ↑ EV size (~200 nm)	45
	Non-small Cell Lung Cancer	Human Bronchoalveolar Lavage Fluid	Ultracentrifugation	~50-200 nm	↑ EGFR ↑ KRAS ↑ ALK ↑ MET ↑ LKB1 ↑ PIK3CA ↑ ROS1	46
Infectious Diseases	Enzootic Bovine Leukosis	Bovine Milk	Successive filtration (1.0, 0.45, and 0.2 μm-pore-size) Ultracentrifugation	~100 nm	↑ TMEM1156 ↑ SRGN ↑ CXCL8 ↑ DEFB4A ↑ FABP5 ↑ LAPM5 ↑ LGALS1 ↑ VIM	153.
Neurological Diseases	Alzheimer's Disease	Human Plasma	-	-	↑ MT-CO3 ↑ MT-ND1 ↑ MT-ND4 ↑ MT-ND2 ↑ MT-ATP6 ↑ MT-ND4 ↑ MT-CYTB ↑ MT-ND ↑ MT-NTP ↑ MT-ND5 ↑ MT-C01 ↑ MT-ND6	63
		Culture media of microglia (HMC3) astrocytoma (1321N1) neuroblastoma (BE(2)-M17) primary embryonic cortical neurons	Ultracentrifugation	~100-300 nm (astrocytes only)		
	Autism Spectrum Disorder	Human Plasma	Precipitation	50-150 nm	↑ *SYT1* loci ↑ *SYT9* loci ↑ *SYP* loci ↓ *SV2C*	71
	Autism Spectrum Disorder	Human Serum	Precipitation L1CAM immuno- pulldown	~40-150 nm	↑ EV concentration ↑ EDNRA ↓ HTR3A	77
	Postpartum Depression	Human Plasma	Membrane affinity spin columns	-	(In third trimester) ↑ CD72 ↑ KYNU	80
					↑ Total mRNA concentration in third trimester, followed by ↓ after birth	
Obstetric Indications	Antenatal Hydronephrosis	Human Amniotic Fluid	Size exclusion chromatography Ultracentrifugation	~75-200 nm	↑ Moesin	88

**Table 4:** Therapeutic applications under investigation for mRNA encapsulated EVs.

Indication		Nanoparticle	Cell Source	mRNA cargo	Loading Method	Significance	Source
Cancer	Breast (HER2+)	Extracellular Vesicles	HEK293	HChrR6	Transfection	Demonstrated EV-mediated delivery of functional exogenous mRNA to tumors.	116
	Breast (HER2+)	Extracellular Vesicles	HEK293	HChrR6	Transfection	Loaded EVs with *in vitr*o transcribed mRNA to avoid the use of plasmids.	119
	Breast (HER2+)	Extracellular Vesicles	HEK293	GSDMD-N	Transfection	Induced pyroptosis in cancer cells through EV-targeted mRNA delivery.	121
	Colon, Melanoma	Outer MembraneVesicles	E. coli	EGFP, OVA, or ADPGK	Transformation	Built an OMV-based mRNA delivery platform to elicit antitumor immunity.	123
	Glioma	Exosomes	MSCs	yCD::UPRT	Transduction	Demonstrated tumor cell internalization of exosomes with mRNA for a suicide gene.	117
	Glioma	Exosomes	MEFs, DCs	PTEN	Transfection	Restored tumor-suppressor function utilizing mRNA-containing exosomes.	122
	Glioma	Small Extracellular Vesicles	MEFs, HEK293T	IFN-γ	Transfection	Induced antitumor activities utilizing immunogenic sEVs.	148
	Pancreatic	Extracellular Vesicles	MEFs, MSCs	TP53	Transfection	Suppressed tumor growth and increased survival with mRNA loaded EVs.	149
CardiovascularDisease	Atherosclerosis (Inflammation)	Exosomes	HEK293T	IL-10	Transfection	Controlled inflammation through exosome-based delivery of functional mRNA.	130
	Cerebral ischemia	Exosomes	HEK293	NGF	Transfection	Delivered mRNA loaded exosomes with therapeutic effects to infarcted regions.	136
	Hypercholesterolemia (familial)	Exosomes	AML12	Ldlr	Transfection	Restore LDL receptor expression through exosome-based mRNA delivery.	133
	Hypercholesterolemia	Extracellular Vesicles	HEK293T	Ldlr	Transfection	Proposed strategy to improve loading and release of therapeutic mRNA in EVs.	134
Infectious disease	COVID-19	Extracellular Vesicles	LSCs	GFP	Electroporation	Enhanced pulmonary bioavailability and therapeutic efficacy with lung derived EVs.	115
	COVID-19	Extracellular Vesicles	LSCs	SARS-CoV-2 spike protein	Electroporation	Demonstrated EV-based inhaled mRNA drug-delivery system superior to LNPs.	125
	HIV-1	Exosomes	HEK293T	ZPAMt	Transfection	Epigenetically repressed HIV-1 infection through exosome-mediated mRNA delivery.	127
NeurologicalConditions	Parkinson's disease	Exosomes	HEK293T	Catalase	Transfection	Reduced neurotoxicity and neuroinflammation through exosome-based mRNA delivery.	118
Other	Inflammatory bowel disease	Extracellular Vesicles	HEK293T	GFP, PGC1α, or IL-10	Transfection	Proposed strategy to improve loading of therapeutic mRNA in EVs for gene therapy.	154
	Obesity	Exosomes	HEK293T	Luciferase or PGC1α	Transfection	Enhanced efficacy and minimized off-target effects with exosome-based mRNA delivery.	155
	Obesity	Exosomes	HEK293T	Bmp7	Transfection	Induced white adipose tissue browning though exosome-mediated mRNA delivery.	156
	Photoaged skin	Extracellular Vesicles	nHDFs	COL1A1	Transfection	Achieved EV-mediated intradermal mRNA delivery for protein-replacement therapy.	143
